# *ZFP804A* mutant mice display sex-dependent schizophrenia-like behaviors

**DOI:** 10.1038/s41380-020-00972-4

**Published:** 2020-12-10

**Authors:** Ying Huang, Jing Huang, Qi-Xin Zhou, Chun-Xian Yang, Cui-Ping Yang, Wan-Ying Mei, Lei Zhang, Qiong Zhang, Ling Hu, Yun-Qing Hu, Ning-Ning Song, Sheng-Xi Wu, Lin Xu, Yu-Qiang Ding

**Affiliations:** 1 Key Laboratory of Arrhythmias, Ministry of Education of China, East Hospital, and Department of Anatomy and Neurobiology, Tongji University School of Medicine, Shanghai, 200092 China; 2 Department of Laboratory Animal Science, Fudan University, Shanghai, 200032 China; 3 Department of Neurobiology, School of Basic Medicine, Fourth Military Medical University, Xi’an, 710032 Shaanxi China; 4 Laboratory of Learning and Memory, Kunming Institute of Zoology, Chinese Academy of Sciences, Kunming, 650223 China; 5 State Key Laboratory of Medical Neurobiology and MOE Frontiers Center for Brain Science, Institutes of Brain Science, Fudan University, Shanghai, 200032 China

**Keywords:** Neuroscience, Schizophrenia

## Abstract

Genome-wide association studies uncovered the association of *ZNF804A* (Zinc-finger protein 804A) with schizophrenia (SZ). In vitro data have indicated that *ZNF804A* might exert its biological roles by regulating spine and neurite morphogenesis. However, no in vivo data are available for the role of *ZNF804A* in psychiatric disorders in general, SZ in particular. We generated *ZFP804A* mutant mice, and they showed deficits in contextual fear and spatial memory. We also observed the sensorimotor gating impairment, as revealed by the prepulse inhibition test, but only in female *ZFP804A* mutant mice from the age of 6 months. Notably, the PPI difference between the female mutant and control mice was no longer existed with the administration of Clozapine or after the ovariectomy. Hippocampal long-term potentiation was normal in both genders of the mutant mice. Long-term depression was absent in male mutants, but facilitated in the female mutants. Protein levels of hippocampal serotonin-6 receptor and GABAB1 receptor were increased, while those of cortical dopamine 2 receptor were decreased in the female mutants with no obvious changes in the male mutants. Moreover, the spine density was reduced in the cerebral cortex and hippocampus of the mutant mice. Knockdown of *ZFP804A* impaired the neurite morphogenesis of cortical and hippocampal neurons, while its overexpression enhanced neurite morphogenesis only in the cortical neurons in vitro. Our data collectively support the idea that *ZFP804A/ZNF804A* plays important roles in the cognitive functions and sensorimotor gating, and its dysfunction may contribute to SZ, particularly in the female patients.

## Introduction

Schizophrenia (SZ) is a debilitating mental disorder with a lifetime prevalence rate of about 1% [1]. The SZ symptoms are complex and affect many aspects in thinking, emotional and cognitive functions, the end results of which tends to be a serious disability in most SZ patients. An emerging hypothesis for the cause of SZ posits that epistatic interactions among multiple genes are involved in the complex SZ pathology [2].

ZNF804A belongs to the zinc-finger protein family containing a C2H2-type domain and is expressed in the brain [3–6]. Genome-wide association studies (GWAS) have revealed *ZNF804A* as one of the strongest candidate genes for SZ [4, 7, 8]. Genetic risks of *ZNF804A* variants achieve the genome-wide significance for SZ in multiple population samples [7, 9–13]. Thus, *ZNF804A* has been a subject of intense research in relation to SZ. Association of the *ZNF804A* risk allele with the structure (e.g., cortical thickness, surface area, and cortical volume) and functions of human brain has been reported [14–18]. Imaging genetics studies have demonstrated that healthy carriers of rs1344706 (an intronic SNP site in *ZNF804A*) risk allele exhibit strong gene dosage-dependent changes in the functional coupling of the dorsolateral prefrontal cortex across the hemispheres and hippocampus, the observation which parallels the similar findings in SZ patients [14, 19]. The dis-connectivity of the left hippocampal formation-posterior cingulate cortex tract seems to be a plausible intermediate phenotype that links rs1344706 with SZ [17]. In addition, *ZNF804A* rs1344706 may be associated with deficits in episodic and working memory [20–22], as well as in the cognitive function according to the data from combining polygenic scores with the risk variants in SZ patients [23].

The biological roles of *ZNF804A* in the nervous system have been explored by many laboratories. Gene ontology analysis of differentially expressed genes has shown a significant effect of *ZNF804A* knockdown on the expression of the genes involved in cell adhesion, suggesting that *ZNF804A* might be involved in processes such as neuronal migration, neurite outgrowth, and synapse formation [24]. Several studies on *ZNF804A* and the mouse homolog *ZFP804A* have demonstrated that these proteins are localized in synapses and participated in neurite formation and dendritic spine development in vitro [25–27]. A recent study showed that *ZFP804A* is required for neural progenitor proliferation and neuronal migration, and overexpression of the gene encoding neurogranin, which is another SZ risk gene and a target of *ZFP804A*, was able to counteract the migration defects caused by *ZFP804A* knockdown [6]. A *ZNF804A* splice variant was also identified, and its expression levels were reduced and associated with the rs1344706 allele in SZ patients [4]. Empirical work and predictive bioinformatic analyses suggest that *ZNF804A* polymorphisms might be able to change the *ZNF804A* expression level or particular isoforms by altering its affinity for DNA/RNA-binding proteins [28].

Significant differences can be seen between sexes in several clinical and biological aspects of SZ. For example, male SZ patients tend to show more severe symptoms, worse cognitive function, poorer treatment response, and generally worse prognosis than female SZ patients do [29, 30]. These sex differences are also evident in the core symptoms: female SZ patients have more affective symptoms while the male patients have more negative symptoms [29–31]. In addition, a female-specific association has been observed between a certain ZN*F804A* variant and SZ [32, 33].

While there has been an abundance of evidences for the genetic association between *ZNF804A* and SZ [34], the mechanism of how the defects in this gene causes SZ has not been understood. In particular, a scant in vivo evidence exists for its biological roles at behavioral level. To understand the biological functions of *ZNF804A*, we generated *ZFP804A* mutant mice. Our results show that the body weight, gross brain architecture, locomotion, and social memory are normal, but the contextual fear and spatial memory are impaired in the mutant mice. We also observed deficit in the sensorimotor gating, but this defect was found only in the female mutant mice in an age-dependent way. Our results provide new insight into the role of *ZFP804A* and, by implication, human *ZNP804A* in cognition and sensorimotor gating, relevant to the core symptoms of SZ.

## Materials and methods

### Histological analysis, immunohistochemistry, and in situ hybridization (ISH)

Animal brain slices, Nissl staining, immunohistochemistry, and ISH were performed as previously described [35]. Briefly, the mice at various desired stages were perfused with 4% paraformaldehyde (PFA) in 0.01-M phosphate-buffered saline (PBS; pH 7.4). PFA-fixed brains were sectioned in the coronal plane on a cryostat after cryoprotection with 30% sucrose in PBS. For Nissl staining, sections were stained with Cresyl Violet. ISH was performed using digoxigenin (DIG)-labeled riboprobes to detect expression patterns of *ZFP804A*, *Cut homeodomain transcription factor 2* (*Cux2*), *Dickkopf homolog* 3 (*Dkk3*), *PlexinD1* (*Plxd1*)*, glutamic acid decarboxylase 67* (*GAD67*), and *vesicular glutamate transporter 1* (*VGLUT1*). The signals were visualized in alkaline phosphatase buffer containing NBT and BCIP. These probes were constructed according to the descriptions on the website of the Allen Brain Atlas (http://www.brain-map.org).

To detect *ZFP804A* transcripts and anti-neuronal nuclei (NeuN) proteins simultaneously, fluorescent in situ hybridization (FISH) and immunofluorescence techniques were combined according to previous reports with minor modifications [36]. To detect the expression of *ZFP804A* in GABAergic neurons, we used GAD67-green fluorescent protein (GFP) knock-in mice, in which GABAergic neurons are revealed by GFP [37]. Briefly, after the hybridization with DIG-labeled *ZFP804A* probes, sections were incubated overnight with a mixture of POD-conjugated anti-DIG antibody (1:3000, #11093274910, Roche Diagnostics) and mouse NeuN (1:5000, #MAB377, Chemicon) or 1-μg/ml affinity purified guinea pig antibody against heat-denatured GFP [38]. The sections were then incubated with TSA-Biotin (1:100; NEN Life Science Products) in 1× Amplification Diluent for 10 min at room temperature. Secondary antibodies included Alexa Fluor 594-conjugated streptavidin (2 μg/mL, #S11227, Invitrogen) for detection of *ZFP804A* mRNA and Alexa Fluor 488-conjugated donkey anti-mouse IgG (4 μg/mL, #R37114, Invitrogen) for visualization of NeuN immunoreactivity, and Alexa Fluor 488-conjugated donkey anti-guinea pig IgG (4 μg/ml, #706-546-148, Jackson Immunoresearch) for visualization of GFP. After being rinsed with 0.01-M PBS (pH 7.4), the sections were counterstained with 4, 6-diamidino-2-phenylindole (DAPI) (1:2000, #D1306, Invitrogen), and were examined and photographed under a bright-field microscope (AH-3/Ni-U, Olympus/Nikon) or a confocal laser scanning microscope (FV-1000, Olympus). All images were imported into Adobe Photoshop CS5 (Adobe Systems Inc.) and only minor adjustments to the contrast and for brightness settings were applied if necessary. The total number of positive neurons in each layer of the cerebral cortex was counted manually from six sections randomly selected from three different animals, and layer distribution of *ZFP804A-*positive neurons was expressed as a percentage of the positive cells of the total cell population.

### Double FISH for detection of *ZFP804A* and *VGLUT1*

The double FISH procedure was carried out as reported previously [39]. Sections were hybridized with a mixture of DIG-labeled *ZFP804A* riboprobe and FITC-labeled riboprobe for *VGLUT1*. Briefly, the hybridized sections were incubated overnight with peroxidase-conjugated anti-DIG sheep antibody and incubated with AP-conjugated streptavidin. The sections were further incubated overnight with peroxidase-conjugated anti-FITC sheep antibody (11-426-346-910, Roche Diagnostics), treated with 1:100 diluted tyramide signal amplification (TSA) plus dinitrophenyl (DNP) kit (NEL747A, PerkinElmer) for 30 min, and incubated with 10-μg/ml AlexaFluor488-conjugated anti-DNP rabbit antibody (A-11097, Invitrogen) for 2 h, to visualize the signals for *VGLUT1* mRNA. Subsequently, the sections were processed with the 2-hydroxy-3-naphtoic acid-2-phenylanilide phosphate (HNPP) Fluorescence Detection kit (HNPP/FastRed TR, 11-758-888-001, Roche Diagnostics) for 3–4 h, to detect the signals for *ZFP804A* mRNA. When the anti-DIG or anti-FITC antibody was omitted as controls, no fluorescence signals were detected.

### Generation of ZFP804A mutant mice

The TALEN-coding *ZFP804A* plasmid was purchased from Shanghai Taiting Biotechnology Co. Ltd, China. Procedures for superovulation, oocytes collection, TALEN injection, and embryo transfer were then carried out as previously described [40]. Briefly, TALEN plasmid was digested by NotI restriction endonuclease. One microgram of the digested plasmid was used as a template for the in vitro transcription reaction using the mMESSAGE mMACHINE SP6 Transcription Kit (Ambion), and then polyadenylated by using a Poly(A) tailing kit (Ambion). Two TALEN mRNAs (1:1 ratio) were diluted with injection buffer (10-mM TrisHCl/0.1-mM EDTA, pH 7.4) at 10 ng/μl. We then carried out microinjection of the mixture into cytoplasm of pronuclear stage oocytes under standard procedures using oocytes obtained from superovulated (C57BL/6 × FVB/N) F1 mice mated with male mice of the same strain. The injected oocytes were transferred into pseudopregnant ICR female mice.

### Genotyping and maintenance of mice

For genotyping of *ZFP804A* mutant mice, genomic DNA was extracted from mouse tail tips. Two PCR reactions were carried out sequentially. The PCR product (size: 820 bp) from the first PCR was used as the amplification template for the second PCR. The primers sequences used for the first PCR were: *ZFP804A*-F1 (5′-atg tac tcc tct gac agc tct c-3′), *ZFP804A*-R1 (5′-tcc cac gcc cat cag ata aat a-3′). The primers sequences used for the second PCR were: *ZFP804A*-F2 (5′-taa taa cac tca tgc ctg cca g-3′), *ZFP804A*-R2 (5′-cta aag ctc cca aat cca tcc g-3′). The second PCR products (size: 680 bp) were sent out for Sanger sequencing with primer *ZFP804A*-F2 or treated with NotI restriction endonuclease. Depend on the sequence integrity, the expected size of homozygous mutant and wild-type digestion were 680 bp, and 250 + 430 bp, respectively.

*ZFP804A* heterozygous mutants were crossed with C57BL/6 wild-type mice for at least four generations, followed by heterozygous × heterozygous brother–sister mating to generate offspring with C57B6/FVB mixed genetic background for all the experiments in this study. In each set of experiments, at least three mutant mice and three or more control mice including WT mice or heterozygous mice were used. Animals were housed at a constant temperature of 23 °C with a 12-h light/dark cycle with free access to food and water. Mice were housed in genotype groups of four to five mice per cage. All experiments on animals have been reviewed and approved by the Animal Research Committee of Tongji University School of Medicine, China.

### Quantitative real-time PCR (qRT-PCR)

Mice at 5-day old were anesthetized on ice, then brains were removed and the desired tissues were dissected out. Total RNA was extracted from each sample with TRIzol (TaKaRa Biotechnology), and 2 μg of the RNA sample was converted to cDNA via the PrimeScript™ RT reagent Kit (TaKaRa Biotechnology). qRT-PCR was performed in triplicate for each sample using ABI-Q7 (Applied Biosystems) with SYBR Green Premix Taq (Qiagen). The resulting cDNA was amplified by an initial denaturation at 95 °C for 5 min, followed by 40 cycles of denaturation at 95 °C for 15 s and 60 °C for 30 s. The primer sequences were: ZFP804A-F (5′-gcg gct gcc cca tgg agt gtt-3′), ZFP804A-R (5′-ttc tcc ttt tca gca tag tc-3′), GAPDH-F (5′-agg tcg gtg tga acg gat ttg-3′), and GAPDH-R (5′-ggg gtc gtt gat ggc aac a-3′).

### Behavioral tests

All behavioral experiments were performed in the light phase, between 0700 and 1800 h in a sound-proof room with a neutral environment. All mice were given a 60-min habituation time after transfer to the behavioral test room. The experimenter was blind to the group identity of the tested mice.

#### Open-field test

The open-field test was carried out in a computer-operated detecting and analysis apparatus (Med Associates). Mice were tested at the age of 2 months. Each mouse was placed in the field of 27.31 × 27.31 cm (*L* × *W*) with a dimly lit background for 30-min of spontaneous exploration. The field floor was divided into nine squares. Average velocity, total distance traveled in the whole arena, and time spent in the central arena were recorded.

#### Three-chamber social interaction test

Sociability and social novelty test were performed on mice as previously described [41], with minor modifications. Both stranger 1 and stranger 2 were wild-type C57B6 mice with matched age, body weight, and sex to the mice being tested. The social test apparatus was made of a clear plexiglass box (90 × 50 × 30 cm, *L* × *W* × *H*) with three equally divided chambers (30 × 50 × 30 cm each). The chambers were interconnected with 5 × 5-cm openings, which could be opened or closed manually. The inverted cylindrical wire cups, which contain the stranger mouse or an object (ping-pong ball), were 10 cm in height and contained a 10-cm floor with the metal bars spaced 0.8 cm apart. A weighted water bottle was placed on each top of the inverted wire cup to prevent the mice from escaping. The day before the test, each of the stranger mice was habituated inside the inverted wire cups, and each of the test mice was habituated to the apparatus with two empty wire cups inside the box for 15 min. On the test day, during the habituation phase, an empty wire cup was placed into the left and right chamber, the test mouse was placed into the center chamber and allowed to explore for 10 min, with all doors open between chambers. During the sociability test phase, an unfamiliar mouse (S1) was placed inside the inverted wire cup in one of the side chambers, an object (O) was placed inside the inverted wire cup on another side chamber, and the test mouse was introduced to the center chamber with the doors to both side chambers closed. Then the doors between chambers were lifted simultaneously, and the test mouse was allowed to explore all three chambers for 10 min. During the social novelty test phase, the test mouse was placed in the central chamber with all doors closed between chambers. After a novel mouse (S2) was introduced in the inverted wired cup, replacing the object (O) in one of the side chambers, the doors between chambers were lifted simultaneously, and the test mouse was allowed to explore all three chambers for an additional 10 min. Time spent in close proximity to the empty cup (E1, E2) or the stranger mice (S1, S2) or object (O) was analyzed. The animals used in this test were previously tested in the open-field assay with a 7-day break in between.

#### Contextual fear conditioning test

The procedures for contextual fear conditioning were similar to those previously described [42], with minor modifications. Mice were placed in a box and received five foot-shocks (1.2 mA, 2 s) with intershock intervals of 2 min (Freeze Frame, Coulbourn Instruments). Freezing behavior was measured as the amount of time mice exhibited freezing behavior during each intershock interval. Then mice were placed back in the box (fear context) 60 min, 1 day, 7 days, and 1 month after fear conditioning and their contextual freezing behavior was measured for 11 min without any foot shocks applied. The animals used in this test were previously tested in the three-chamber social interaction assay with a 7-day break in between.

#### Prepulse inhibition (PPI) test

PPI tests were performed as previously described [43], with minor modifications. Experiments were performed with sound attenuating test chambers (65 × 35 × 25 cm, *L* × *W* × *H*). Each chamber was equipped within a commercial startle reflex system (SR Lab). Each test session began with a 5-min acclimation period in the presence of 50-dB acoustic background noise followed by twelve 100-dB startle pulse (20 ms). On the subsequent 48 trails, the startle tone was either presented alone or after three levels of prepulse intensity (65, 72, and 83 dB, 20 ms) with a delay of 100 ms in a randomized order. The average intertrial between each trial was 30 s (range: 20–40 s). The average startle amplitude during the 100 ms following the onset of each startle stimulus was automatically recorded.

Percentage of prepulse inhibition was calculated for each mouse at each prepulse stimulus intensity using the equation: PPI = 100 − [(prepulse/startle alone) × 100], where prepulse is the average startle response on trials in which there was a prepulse stimulus and startle alone is the average startle response on the trials in which the only startle stimulus was presented (excluding the first 12 trials of the session).

To study the effect of antipsychotic drugs on the aberrant prepulse inhibition ability in *ZFP804A* mutant mice, independent cohorts of *ZFP804A* mutant mice at the age of 6 months received Clozapine (1-mg/kg i.p., MedChemExpress) or saline on 2 consecutive days. PPI tests were performed 30 min after the second administration. To explore the estrogen effect on the aberrant prepulse inhibition ability in *ZFP804A* mutant mice, ovariectomy was performed on independent cohorts of 6-month-old female *ZFP804A* mutant and control mice, then PPI tests were conducted when these mice were 8-month old.

#### Morris water maze test

To assess spatial learning and memory, the Morris water maze test was performed as described previously [44], with minor modifications. The blue circular pool was 120 cm in diameter and was divided into four equal quadrants with two hypothetical crossed lines. The hidden circular platform located in the middle of the target quadrant was 10 cm in diameter, and submerged 1 cm below the water surface. During the training period, mice were trained to find the hidden platform over 8 consecutive days with four trials per day using a semi-random set of start locations, with the restriction that one trial each day was from each of the four different starting positions. If a mouse failed to find the platform within 60 s, it was picked up and placed on the platform for 20 s. On the 9th day, 24 h after the last training session, the platform was removed, and 60 s were given to each mouse to search for the platform in the pool, with the starting location opposite to the previous position. The movement of the mice was monitored using Noldus software (EthoVision XT 8.0, Noldus Technology). Escape latency to find the platform, total distance moved, average velocity, total distance to platform, and duration in platform zone were automatically analyzed by the software. The animals used in this test previously underwent the PPI test with a 7-day break between the tests.

### Electrophysiological recording

Two-month old mice were euthanized via CO_2_ and subjected to electrophysiological studies as previously described [42], with minor modifications. Briefly, hippocampal coronal slices (350 μm) were obtained using a vibratome (Leica VT 1000S) in ice-cold artificial CSF (ACSF) [in mM]: 120 sucrose, 1.25 NaH_2_PO_4_, 2.5 KCl, 8 MgSO_4_, 0.5 CaCl_2_, 26 NaHCO_3_, and 10 D-glucose and gassed with 95% O_2_ and 5% CO_2_. The slices were maintained in an incubation chamber for 0.5 h in 300 ml of ACSF heated to 30 ± 1 °C and then maintained at room temperature (22–25 °C) with Artificial ACSF [in mM]: 120 NaCl, 1.25 NaH_2_PO_4_, 2.5 KCl, 2 MgSO_4_, 2 CaCl_2_, 26 NaHCO_3_, and 10 D-glucose, saturated with 95% O_2_ and 5% CO_2_ for at least 1 h at room temperature (22–25 °C) before recording. Then slices were placed in a recording chamber and perfused by ACSF with a flow rate of 3–5 ml/min. A glass recording-electrode filled with ACSF (1–2 MΩ) was placed in the stratum radiatum of the CA1 region. The field EPSPs (fEPSPs) were evoked by stimulation of the Schaffer collaterals. Electrical stimuli were delivered at a frequency of 0.033 Hz to record baseline fEPSP and at a stimulation intensity adjusted to give an fEPSP amplitude of 50% maximum response. In all experiments, baseline of synaptic transmission was monitored for 30 min before the stimulation. Long-term potentiation (LTP) was induced by high-frequency stimulation (HFS, 100 pulses at 200 Hz, 3 trains, 20-s intertrain intervals). Long-term depression (LTD) was induced by low-frequency stimulation (LFS, 900 pulses at 1 Hz, 1 train). All data were recorded using pCLAMP10 software (Molecular Devices). The fEPSPs were analyzed using Clampfit 10.3 software (Molecular Devices).

### Preparation, Western blotting, and quantification of protein samples from mouse brain

Cortical and hippocampal tissues were prepared from 6-month-old mice. Briefly, desired regions were dissected and lysed in M-PER reagent using a protease inhibitor cocktail (Thermo Scientific). Equal amounts of unboiled proteins were fractionated by SDS-PAGE, transferred to nitrocellulose membranes, and blotted with primary antibodies. The following primary antibodies were used: rat anti-D1R (1:500, #D2944, Sigma), rabbit anti-D2R (1:300, #55084-1-AP, Proteintech), rabbit anti-D5R (1:1000, #ab181623, Abcam), rabbit anti-5-HT1bR (1:100, #ab13869, Abcam), rabbit anti-5-HT2aR (1:100, #24288, Immuno Star), rabbit anti-5-HT2cR (1:100, #24505, Immuno Star), rabbit anti-5-HT3aR (1:500, #NB100-56351, NOVUS), rabbit anti-5-HT6R (1:500, #24507, Immuno Star), rabbit anti-5-HT7R (1:200, #13830-1-AP, Proteintech), rabbit anti-GABAB1R (1:1000, #ab16604, Abcam), rabbit anti-GABAB2R (1:1000, #ab75838, Abcam), and mouse anti-GAPDH (1:2000, #LF206, EpiZyme) or rabbit anti-β-Tubulin (1:1000, #LF202, EpiZyme) was used as an internal control. After primary antibody incubation, the membranes were treated with an HRP goat anti-rat (IgG) secondary antibody (1:1000, #ab97057, Abcam), or an HRP goat anti-mouse (IgG) secondary antibody (1:1000, #LF101, EpiZyme), or an HRP goat anti-rabbit (IgG) secondary antibody (1:1000, #LF102, EpiZyme) and exposed for chemiluminescent detection (Thermo Scientific). The protein levels were analyzed using Adobe Photoshop CS5 (Adobe Systems) and normalized to the internal control. The values obtained for the mutant mice were then normalized to those of control mice. Statistical significance was tested using the one-sample *t*-test for normalized values by testing whether the measured value differed significantly from the hypothetical value 1.0 (i.e., control mice levels).

### Golgi staining and dendritic spine counting

Golgi staining and spine counting was performed as previously described [41], with minor modifications. Three pairs of 8-month-old female mice were used for dendritic spine counting. Standard commercial kit (FD Rapid Golgi Stain Kit) was used for mouse brain Golgi staining. Images of 150-μm-thick coronal cryostated slices were taken using a microscope (Precipoint M8). Then the Z-stack images were merged using Adobe Photoshop (Adobe Systems Incorporated). To quantify the spine density, at least twelve neurons were taken respectively in the layer II/III of frontal associated cortical area and the CA1 hippocampal area of each mouse brain. The spine density of secondary or tertiary dendritic branches was counted.

### Primary neuronal culture, immunocytochemistry, and dendritic branching analysis

Cortical and hippocampal cultures were prepared from embryonic day 18.5 embryos or new-born mice as described previously [45], with minor modifications. Cells were plated onto 14/18-mm glass coverslips (Neuvitro), at a density of 1–2 × 10^5^ cells/well. Neurons were cultured in maintenance medium: neurobasal medium (#21103049, Life technology) supplemented with 2% B27 (#17504044), 0.5-M GlutaMAX (#35050061, Life technology), and 1% Penicillin/Streptomycin (#15140122, Life technology). Neuron cultures were maintained in presence of 200-μM D, L-APV beginning on differentiation in vitro (DIV) day 4. Half media changes were performed twice a week until the desired age (DIV 23).

Hippocampal cells were fixed in 4% PFA, permeabilized with 0.1% triton-100, and blocked in 10% BSA w/PBS. Cells were then co-incubated with rabbit anti-ZNF804A (1:500, #GTX121178, Genetex) and mouse anti-MAP2 (1:500, #ab11267, Abcam), or mouse anti-PSD95 (1:500, #MA1-046, Thermofisher) and rabbit anti-MAP2 (1:200, #ab32454, Abcam) at 4 °C overnight. Immunoreactive signals were visualized by incubating cells with a mixture of Alexa Fluor 488 donkey anti-mouse IgG (1:500, #A21202, Invitrogen) and biotinylated horse anti-rabbit IgG (1:500, #BA1100, Jackson Immunoresearch), or Alexa Fluor 488 donkey anti-rabbit IgG (1:500, #A21206, Invitrogen) and biotinylated horse anti-mouse IgG (1:500, #BA2000, Jackson Immunoresearch) for 3 h, followed by incubation with Cy3-conjugated streptavidin (1:1000, #016-160-084, Jackson Immunoresearch).

Images were obtained on an epifluorescent microscope (Nikon Ni-U, Japan) equipped with a Coolpix digital camera (Nikon DS-Fi3). All images were imported into Adobe Photoshop (Adobe Systems Inc.) and only minor adjustments to the contrast and brightness settings were applied if necessary.

To quantify dendritic branching or positive immunostaining signals in vitro, circles or traces were drawn using NIS-ELEMENTS software (Nikon) or using the Sholl analysis tool in ImageJ software. We then counted the numbers of the dendrites of a neuron intersected with the circumference circles drawn around the neuronal soma or immunoreactive signals at every 10-μm intervals along the dendrites (*n* ≥ 35 for each group from three cultures).

### HEK293 cell cultures, transfection, and Western blotting

HEK293 cells (#D611031, Sangon, mycoplasma free) were cultured in Dulbecco’s modified Eagle’s medium (Gibco) supplemented with 10% fetal bovine serum (Hyclone). At 80% confluence, cells were transfected (for details, see “Results”) using lipofectamine 2000 (Invitrogen). Twenty-four hours later, cells were lysed in ice-cold RIPA buffer containing 150-mM NaCl, 30-mM HEPES, 100-mM NaF, 1% Triton-X 100, and 0.01% SDS. Samples were then loaded on SDS-PAGE, transferred to a membrane filter, and then probed with mouse rabbit anti-ZNF804A (1:500, #SB2702531, Sigma) or rabbit anti-β-tubulin (1:1000, #LF202, EpiZyme). The patterns were developed with an HRP goat anti-rabbit (IgG) secondary antibody (1:1000, #LF102, EpiZyme) and visualized with enhanced chemiluminescence (Pierce).

The sequences of the *ZFP804A*-short hairpin RNA (shRNA) constructs were targeted against sequences of mouse *ZNF804A* messenger RNA (NM_175513.3). The sense sequences of these shRNAs are: shRNA#1 GCT CTG AGG AGA AAG GTA ACT; shRNA#2 GCA ACG GAC ACT TTC GCA ACA; shRNA #3GCG AGT AAC TGC ACA GCA AAT. The complementary oligonucleotide was annealed and inserted into the pSUPER vector (OligoEngine). CAG-*ZFP804A* was generated by cloning mouse *ZFP804A* cDNA into the pCAGGS vector. The shRNA#2 or CAG-*ZFP804A* was used to transfect into isolated cortical and hippocampal neurons derived from new-born wild-type mice at DIV1, then cultured for 2 days for further analysis.

### Statistical analysis

To do the quantitation analysis, the assessors were blind to the genotype of the mice. Statistical analyses were performed using GraphPad Prism 8 software. All values were expressed as the mean ± SEM. Comparisons were made using Student’s *t*-test, or one-way ANOVA, or two-way ANOVA with Bonferroni correction or two-way repeated measures ANOVA with Bonferroni correction as mentioned in each figure legend. Results were considered significant when *P* value < 0.05. The number of samples indicates biological replicates and also are indicated in each figure legend.

## Results

### *ZFP804A* expression in the cerebral cortex and hippocampus of adult mice

**Fig. 1:**
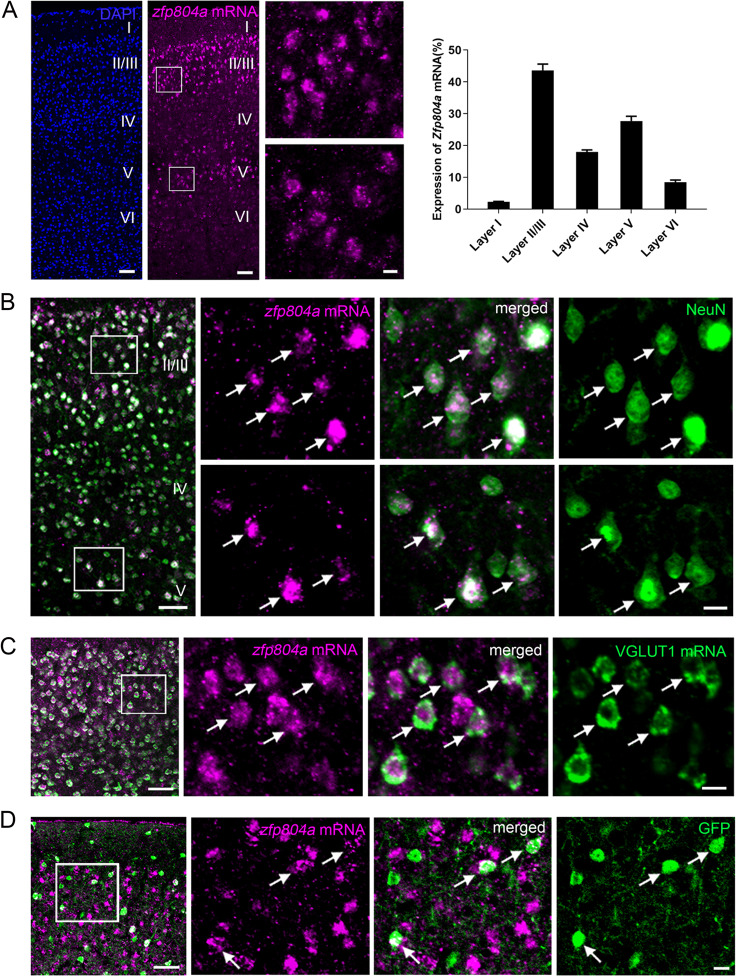
Fluorescent ISH shows the distribution of *ZFP804A* mRNA in the cerebral cortex of the adult mouse. **A***ZFP804A* mRNA (purple, pseudo-color) are primarily located in layers II–IV and V, and to a less degree in layers IV and VI. Sections are counterstained with DAPI to show cortical layers. Higher magnification views of the boxed areas illustrate the location of *ZFP804A* mRNA in the cells of layers II/III and layer V, respectively, and the graph shows the fluorescence intensities (*ZFP804A* mRNA) in cortical layers. Data are means ± standard error of the mean (SEM), *n* = 3. **B** A combination of in situ hybridization of *ZFP804A* (purple, pseudo-color) and NeuN immunostaining (green) shows almost all the *ZFP804A*-expressing cells are immunoreactive for NeuN. Higher magnification views of the boxed areas show the co-localization of *ZFP804A* mRNA and NeuN in layers II/III and layer V, respectively. Arrows indicate the double-labeled neurons. **C** Double labeling for *ZFP804A* mRNA (purple, pseudo-color) and VGLUT1 mRNA (green) in the cerebral cortex. A higher magnification view of the boxed area shows the co-localization of *ZFP804A* mRNA and VGLUT1 in layers II/III. Arrows indicate part of the double-labeled neurons. **D** Double labeling for *ZFP804A* mRNA (purple, pseudo-color) and GFP protein (green) in the cerebral cortex in GAD67-GFP knock-in mice. A higher magnification view of the boxed area shows the co-localization of *ZFP804A* mRNA and GFP in layers II/III. Arrows indicate the double-labeled neurons. Scale bars = 50 μm (left and middle panels in **A**, left panel in **B**–**D**), and 10 μm (right two magnified panels in **A**; right six magnified panels in **B**; right three magnified panels in **C** and **D**).

We used FISH to investigate the expression pattern of *ZFP804A* in the cerebral cortex and hippocampus. The results showed that *ZFP804A* mRNA was distributed throughout the cortex. Among the cortical layers *ZFP804A*-expressing neurons were more abundantly located in layers II/III and V (Fig. 1A). A combination of FISH of *ZFP804A* with immunostaining of NeuN showed that ~96% of *ZFP804A*-expressing cells were co-immunostained with NeuN antibody (Fig. 1B), showing that *ZFP804A* is primarily expressed by cortical neurons. In addition, intense ISH signals were also observed in hippocampal CA1-3 regions and dentate gyrus (Supplementary Fig. S1A), and nearly all the *ZFP804A-*positive cells were NeuN-positive (Supplementary Fig. S1B).

As a first step toward understanding the roles of *ZFP804A* in regulating brain functions, it is important to determine which neuronal types express *ZFP804A*. We performed double FISH using a *VGLUT1* probe to label excitatory neurons, and combination of FISH and fluorescent immunostaining of GFP in GAD67-GFP knock-in mice to label inhibitory neurons. In the cerebral cortex, *ZFP804A* mRNA was co-localized with *VGLUT1* mRNA, and double-labeled neurons were present in around 77% of *ZFP804A*-expressing neurons (Fig. 1C). Furthermore, *ZFP804A* mRNA was immunostained with GFP antibody in GAD67-GFP knock-in mice and ~23% of *ZFP804A*-positive neurons were co-labeled with GFP (Fig. 1D). Similar results were obtained in the hippocampus (data not shown). Thus, ~3/4 of *ZFP804A*-expressing neurons are excitatory while the rest of them are inhibitory in the cerebral cortex and hippocampus.

### Generation of *ZFP804A* mutant mice

**Fig. 2:**
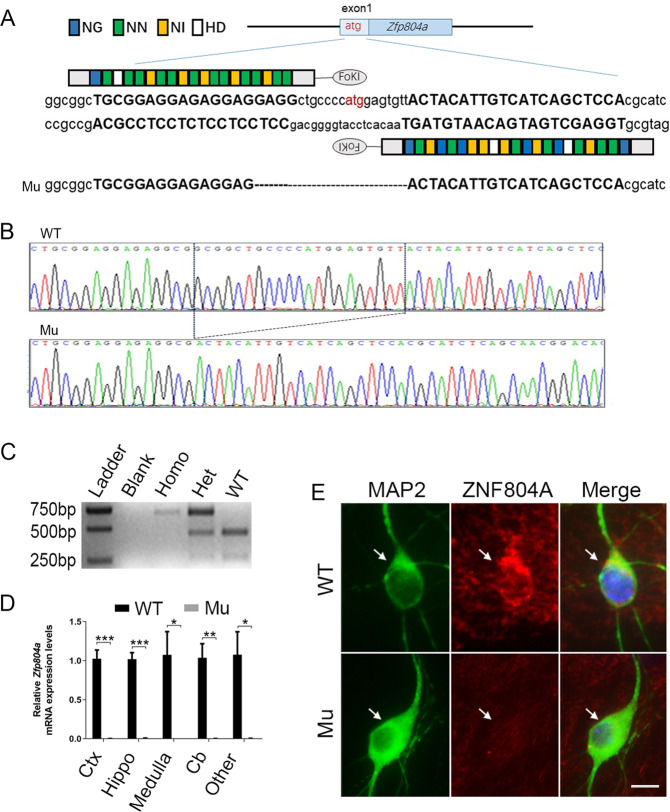
Generation of *ZFP804A* mutant mice via TALENs. **A** Schematic of *ZFP804A* TALEN and its recognition sequence in the ATG area of exon1 of *ZFP804A*. TAL repeats are color-coded to represent each of four repeat variable di-residues (RVDs); each RVD recognizes one corresponding DNA base (NI = A, NG = T, HD = C, NN = G). ATG is indicated in red. Nucleotides bound by TALEN spacer are capitalized. The targeted mutation allele with *ZFP804A* deletion (21-base deletion) is indicated by dotted lines. **B** Sanger sequencing results around the TALEN spacer area of the wild-type (WT) and *ZFP804A* mutant mice. The difference of 21-base between WT and mutant is indicated in the frame. **C** A representative image shows genotyping results after NotI digestion on the second PCR amplification products. DNA fragment lengths of the size markers are shown on the left. Homo homozygote, Het heterozygote. **D** *ZFP804A* mRNA levels are undetectable in indicated tissues of *ZFP804A* mutant mice at P5 compared with age-matched WT controls (Student’s *t* test, *t*_*Ctx*_ = 10.15, *P*_*Ctx*_ < 0.0001***; *t*_Hippo_ = 12.17, *P*_Hippo_ < 0.0001***; *t*_Medulla_ = 3.592, *P*_Medulla_ = 0.022*; *t*_*Cb*_ = 5.726, *P*_*Cb*_ = 0.004**; *t*_Other_ = 3.642, *P*_Other_ = 0.021*). *n* = 3–6 in each group. * *P* < 0.05, **P < 0.01; ***P < 0.001. WT control wild-type mice, Mu *ZFP804A* mutant mice, Ctx cerebral cortex, Hippo hippocampus, Cb cerebrum. **E** Representative images of *ZFP804A* immunostaining in hippocampal cultured neurons at DIV 23 from WT control and mutant mice. Expression of ZNF804A is undetectable in MAP2-positive neurons (arrows) of *ZFP804A* mutant mice compared with WT controls. Scale bar = 10 μm.

To generate *ZFP804A* mutant mouse, we used TALEN-mediated gene editing [40]. TALEN recognition sequence was set at the initiation codon area of exon1 of *ZFP804A* (Fig. 2A), so that ZFP804A protein would be truncated owing to a frameshift mutation once the TALENs caused an indel mutation. F_0_ mice were obtained after microinjection of TALENs into fertilized eggs. Mutations of *ZFP804A* were verified through Sanger sequencing of PCR-based genomic DNA and/or NotI cleavage (Fig. 2B, C), and further confirmed by qRT-PCR (Fig. 2D), as well as by immunostaining in cultured hippocampal and cortical neurons from *ZFP804A* mutant mice (Fig. 2E, and data not shown). Homozygous *ZFP804A* mutant mice are viable and fertile; the growth rate and body weight are also normal (data not shown).

### Hippocampus-dependent memories are impaired in *ZFP804A* mutant mice

We carried out behavioral tests to explore whether *ZFP804A* mutation affected the behaviors including locomotion, social memory, and the hippocampus-dependent spatial and fear memory. Both male and female adult mice (2–4 months old) were investigated.

**Fig. 3:**
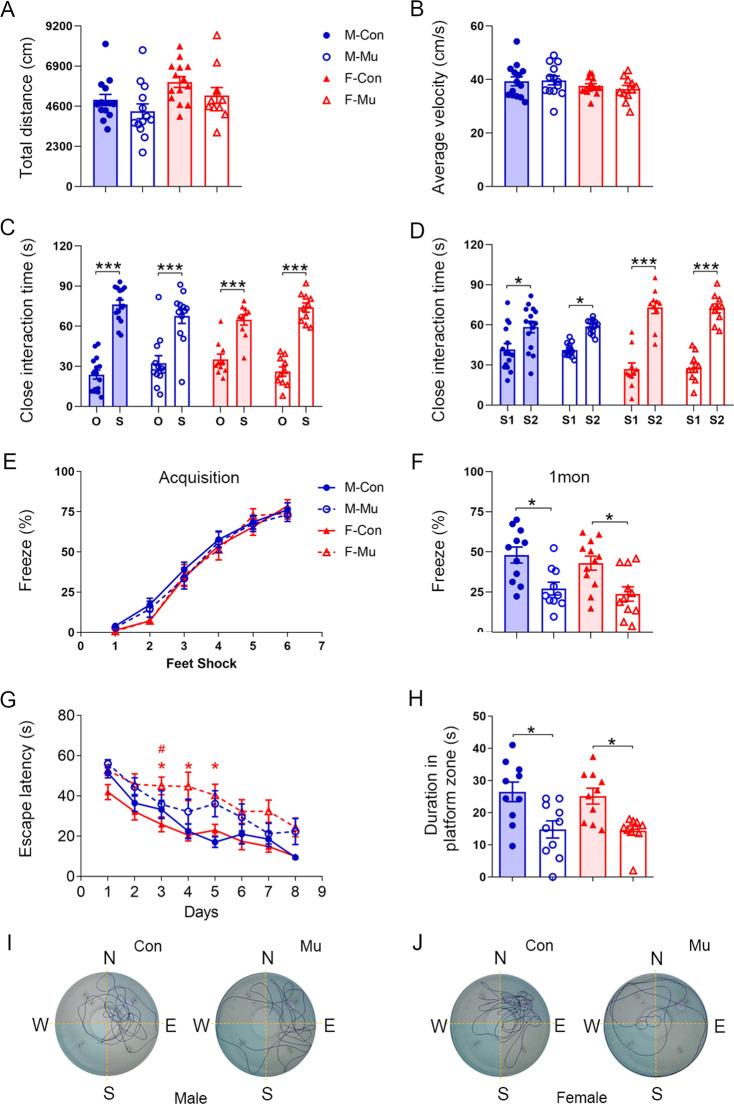
Deficits in contextual fear and spatial memory in *ZFP804A* mutant mice. **A** Total travel distance in the open-field test. No significant differences are observed between control and *ZFP804A* mutant mice. *n* = 11–14 in each group. Two-way ANOVA with Bonferroni correction analysis. Genotyping effect: *F* [1, 48] = 3.579, *P* = 0.0646; sex effect: *F* [1, 48] = 6.605, *P* = 0.0133*; interaction: *F* [1, 48] = 0.02646, *P* = 0.8717. **B** Average velocity in the open-field test. No significant differences are observed between control and *ZFP804A* mutant mice. *n* = 11–14 in each group. Two-way ANOVA with Bonferroni correction analysis. Genotyping effect: *F* [1, 48] = 0.0934, *P* = 0.7612; sex effect: *F* [1, 48] = 3.018, *P* = 0.0888; interaction: *F* [1, 48] = 0.2692, *P* = 0.6063. **C** Time spent on close interaction with an object (O) versus stranger mouse (S) in the phase-II social interaction test. Mice from each group all spend a longer time with S than O (*P*_*M-C*_ = 0.037*; *P*_*M-Mu*_ < 0.0001***; *P*_*F-C*_ < 0.0001***; *P*_*F-Mu*_ < 0.0001***). *n* = 10–15 in each group. Two-way ANOVA with Bonferroni correction analysis. Genotyping and sex effect: *F* [3, 86] = 2.193 × 10^−14^, *P* > 0.999; object and subject effect: *F* [1, 86] = 188.4, *P* < 0.0001***; interaction: *F* [3, 86] = 3.300, *P* = 0.024*. **D** Time spent on close interaction with a familiar mouse (S1) versus stranger mouse (S2) in the phase-III social interaction test. Mice from each group all spend a longer time with S2 than S1 (*P*_*M-C*_ < 0.0159*; *P*_*M-Mu*_ < 0.0318*; *P*_*F-C*_ < 0.0001***; *P*_*F-Mu*_ < 0.0001***). *n* = 10–15 in each group. Two-way ANOVA with Bonferroni correction analysis. Genotyping and sex effect: *F* [3, 86] = 1.758 × 10^−^^15^, *P* > 0.999; subjects effect: *F* [1, 86] = 118.8, *P* < 0.0001***; interaction: *F* [3, 86] = 9.449, *P* < 0.0001***. **E** Freezing behaviors provoked by foot shocks in the contextual fear conditioning are similar between control and ZFP804A mutant mice. *n* = 10–12 in each group. Two-way ANOVA repeated measures with Bonferroni correction analysis. Genotyping and sex effect: *F* [3, 40] = 0.3387, *P* = 0.7974; time effect: *F* [3.823, 152.9] = 297.8, *P* < 0.001***; interaction: *F* [15, 200] = 0.6554, *P* = 0.8257. **F** One month after the contextual fear conditioning, exposure to the environment evokes less freezing behavior in *ZFP804A* mutant mice than in wild-type control mice (*P*_male_ = 0.015*; *P*_female_ = 0.022*). *n* = 10–12 in each group. Two-way ANOVA with Bonferroni correction analysis. Genotyping effect: *F* [1, 40] = 19.81, *P* < 0.001***; sex effect: *F* [1, 40] = 0.8612, *P* = 0.359; interaction: *F* [1, 40] = 0.0369, *P* = 0.8486. **G** Escape latencies throughout the 8-d learning trials in the Morris water maze test. *n* = 9–10 in each group. Two-way ANOVA repeated measures with Bonferroni correction analysis. Control and *ZFP804A* mutant mice from male group show similar escape latencies on each day during the trials. Genotyping effect: *F*_male_ [1, 18] = 3.338, *P*_male_ = 0.0843; time effect: *F*_male_ [3.517, 63.31] = 22.99, *P*_male_ < 0.001***; interaction: *F*_male_ [7, 126] = 1.678, *P*_male_ = 0.1202. But mice from female group show significant difference escape latencies on 3 days during the trials between control and *ZFP804A* mutant mice (*P*_*F*-day3_ = 0.0129*; *P*_*F-*day4_ = 0.0338*; *P*_*F-*day5_ = 0.0244*). Genotyping effect: *F*_female_ [1, 19] = 18.81, *P*_female_ = 0.0004***; time effect: *F*_female_ [3.905, 74.2] = 14.86, *P*_female_ < 0.001***; interaction: *F*_female_ [7, 133] = 0.5931, *P*_female_ = 0.7606). Female *ZFP804A* mutant mice also appear lower learning ability comparing to control female mice on day 3 during the learning trials if analyzed with the combination of genotyping, sex, and time effects together (*P*_*F-*day3_ = 0.0107#). Genotyping and sex effect: *F* [3, 34] = 5.909, *P* = 0.0023**; time effect: *F* [4.159, 141.4] = 38.43, *P* < 0.001***; interaction: *F* [21, 238] = 1.072, *P* = 0.3791. **H** During spatial memory retrieval test, impaired spatial memory is evident in *ZFP804A* mutant mice, which stay less time in the platform zoon (*P*_male_ = 0.011*; *P*_female_ = 0.024*). *n* = 9–10 in each group. Two-way ANOVA with Bonferroni correction analysis. Genotyping effect: *F* [1, 36] = 20.59, *P* < 0.001***; sex effect: *F* [1, 36] = 0.1205, *P* = 0.7305; interaction: *F* [1, 36] = 0.0375, *P* = 0.8474. **I** Representative traveling traces of the male control and *ZFP804A* mutant mice in Morris water maze. **J** Representative traveling traces of the female control and *ZFP804A* mutant mice in Morris water maze. E east, W west, N north, S south, M male mice, F female mice, Con wild-type controls, Mu *ZFP804A* mutant mice.

Locomotion was assessed by using the open-field test, and we detected no significant differences in the travel distance or average velocity between control and *ZFP804A* mutant mice (Fig. 3A, B). Social memory was examined by using the three-chamber social interaction test, in which both control and *ZFP804A* mutant mice showed a significant preference for the animated subject (S) over the inanimate object (O) (Fig. 3C) and a similar preference for the new Stranger 2 (S2) over the familiar Stranger 1 (S1) (Fig. 3D). These results show that social memory is not affected in *ZFP804A* mutant mice.

We then examined contextual fear memory in *ZFP804A* mutant mice. During the fear conditioning, we detected no difference in freezing behavior between control and *ZFP804A* mutant mice (Fig. 3E). The short-term memory (1 h after the conditioning) and long-term memory (1 day after the conditioning) were not different between control and *ZFP804A* mutant mice. However, the remote memory, which was examined 7 days and 1 month after the conditioning was significantly decreased in *ZFP804A* mutant mice, compared with controls (Fig. 3F and Supplementary Fig. S2). These results indicate that remote contextual fear memory is impaired in the absence of functional *ZFP804A*.

Spatial learning and memory was examined using the Morris water maze. We observed that, on the day 3–5 during training trials, female *ZFP804A* mutant mice took slightly longer time (escape latencies) in finding the hidden platform than female control mice did. That is, female *ZFP804A* mutant mice displayed a mild deficit in spatial learning. However, male *ZFP804A* mice performed the spatial learning task as proficiently as the male control mice (Fig. 3G). One day after the training for 8 days, *ZFP804A* mutant mice of both genders spent less time in the target zone compared with control mice (Fig. 3H–J), indicating that spatial memory is impaired in the mutant mice. Note that total distance of movement and swimming speed were both similar between mutant and control mice (Supplementary Fig. S3), showing that the motor functions are not affected by *ZFP804A* mutation.

Taken together, these results indicate that remote contextual fear memory and long-term spatial memory are impaired in both genders of *ZFP804A* mutant mice, while spatial learning ability is defective only in female *ZFP804A* mutant mice.

### Deficit sensorimotor gating in female *ZFP804A* mutant mice

**Fig. 4:**
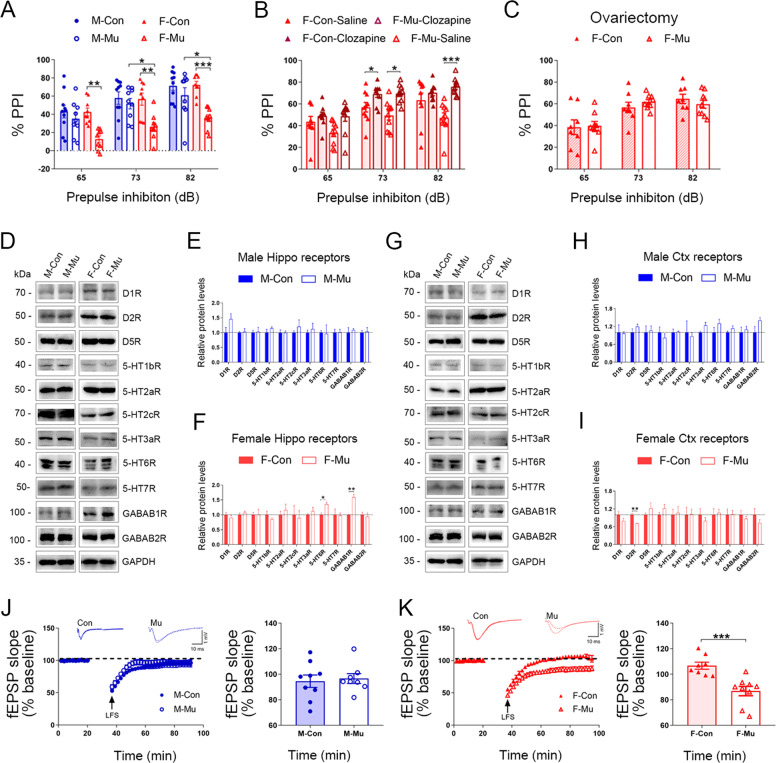
Impaired sensorimotor gating shown by the PPI test in female ZFP804A mutant mice. **A** PPI values for wild-type control and *ZFP804A* mutant mice at three different prepulse intensities (65, 73, and 82 dB) at the age of 8 months. Significantly reduced PPI is observed in female *ZFP804A* mutant mice compared with female controls (*P*_*65dB*_ = 0.0044**; *P*_*73dB*_ = 0.0088**; *P*_*82dB*_ = 0.0004***), and with male *ZFP804A* mutant mice (*P*_*73dB*_ = 0.0397*; *P*_*82dB*_ = 0.0205*). *n* = 9–10 in each group. Two-way ANOVA with Bonferroni correction analysis. At 65 dB, genotyping effect: *F* [1, 33] = 11.35, *P* = 0.0019**; sex effect: *F* [1, 33] = 4.170, *P* = 0.0492*; interaction: *F* [1, 33] = 3.848, *P* = 0.0583. At 73 dB, genotyping effect: *F* [1, 33] = 8.927, *P* = 0.0053**; gender factor: *F* [1, 33] = 4.706, *P* = 0.0374*; interaction: *F* [1, 33] = 3.928, *P* = 0.0559. At 82 dB, genotyping effect: *F* [1, 33] = 17.71, *P* = 0.0001***; sex effect: *F* [1, 33] = 4.658, *P* = 0.0383*; interaction: *F* [1, 33] = 5.574, *P* = 0.0243*. **B** Impaired PPI is restored in female *ZFP804A* mutant mice by the administration of clozapine (*P*_*73dB*_ = 0.0025**; *P*_*82dB*_ = 0.0004***). *n* = 9–10 in each group. One-way ANOVA with Bonferroni correction analysis. At 65 dB, treatment effect: *F* [3, 35] = 3.168, *P* = 0.0363*. At 73 dB, treatment effect: *F* [3, 35] = 7.087, *P* = 0.0008***. At 82 dB, treatment effect: *F* [3, 35] = 7.721, *P* = 0.0004***. **C** PPI values are normal in ovariectomized female *ZFP804A* mutant mice compared with female control mice at three different prepulse intensities (65, 73, and 82 dB) at the age of 8 months. *n* = 8–9 in each group. Student’s *t* test analysis (*t*_*65dB*_ = 0.1673, *P*_*65dB*_ = 0.8693; *t*_*73dB*_ = 0.9923, *P*_*73dB*_ = 0.3368; *t*_*82dB*_ = 0.8783, *P*_*82dB*_ = 0.3936). **D** Representative blots for protein detected by indicated antibodies in the hippocampal tissues from control and *ZFP804A* mutant mice at the age of 6 months. **E** Quantification of relative levels of proteins as normalized to GAPDH or β-Tubulin protein expression from male hippocampus at the age of 6 months. *n* = 3 per protein per genotype. One-sample *t-*test (*t*_*D1R*_ = 1.757, *P*_*D1R*_ = 0.153; *t*_*D2R*_ = 0.265, *P*_*D2R*_ = 0.803; *t*_*D5R*_ = 0.175, *P*_*D5R*_ = 0.868; *t*_*5HT1bR*_ = 1.149, *P*_*5HT1bR*_ = 0.314; *t*_*5HT2aR*_ = 0.015, *P*_*5HT2aR*_ = 0.988; *t*_*5HT2cR*_ = 0.867, *P*_*5HT2cR*_ = 0.434; *t*_*5HT3aR*_ = 0.577, *P*_*5HT3aR*_ = 0.594; *t*_*5HT6R*_ = 0.119, *P*_*5HT6R*_ = 0.91; *t*_*5HT7R*_ = 0.061, *P*_*5HT7R*_ = 0.954; *t*_*GABAB1R*_ = 0.481; *P*_*GABAB1R*_ = 0.655; *t*_*GABAB2R*_ = 0.213, *P*_*GABAB2R*_ = 0.841). **F** Quantification of relative levels of proteins as normalized to GAPDH or β-Tubulin protein expression from female hippocampus at the age of 6 months. *n* = 3 per protein per genotype. One-sample *t-*test (*t*_*D1R*_ = 0.831, *P*_*D1R*_ = 0.452; *t*_*D2R*_ = 0.808, *P*_*D2R*_ = 0.464; *t*_*D5R*_ = 0.05, *P*_*D5R*_ = 0.962; *t*_*5HT1bR*_ = 1.113, *P*_*5HT1bR*_ = 0.328; *t*_*5HT2aR*_ = 0.721, *P*_*5HT2aR*_ = 0.510; *t*_*5HT2cR*_ = 0.272, *P*_*5HT2cR*_ = 0.799; *t*_*5HT3aR*_ = 0.913, *P*_*5HT3aR*_ = 0.412; *t*_*5HT4R*_ = 0.201, *P*_*5HT6R*_ = 0.014*; *t*_*5HT7R*_ = 0.102, *P*_*5HT7R*_ = 0.923; *t*_*GABAB1R*_ = 6.138, *P*_*GABAB1R*_ = 0.003**; *t*_*GABAB2R*_ = 0.4129, *P*_*GABAB2R*_ = 0.700). **G** Representative blots for protein detected by indicated antibodies in the cortical tissues from control and *ZFP804A* mutant mice at the age of 6 months. **H** Quantification of relative levels of proteins as normalized to GAPDH or β-Tubulin protein expression from male cortex at the age of 6 months. *n* = 3 per protein per genotype. One-sample *t-*test (*t*_*D1R*_ = 0.128, *P*_*D1R*_ = 0.903; *t*_*D2R*_ = 1.306, *P*_*D2R*_ = 0.261; *t*_*D5R*_ = 0.236, *P*_*D5R*_ = 0.824; *t*_*5HT1bR*_ = 0.785, *P*_*5HT1bR*_ = 0.476; *t*_*5HT2aR*_ = 0.175, *P*_*5HT2aR*_ = 0.869; *t*_*5HT2cR*_ = 0.317, *P*_*5HT2cR*_ = 0.767; *t*_*5HT3aR*_ = 2.117, *P*_*5HT3aR*_ = 0.101; *t*_*5HT6R*_ = 1.369, *P*_*5HT6R*_ = 0.242; *t*_*5HT7R*_ = 1.280, *P*_*5HT7R*_ = 0.269; *P*_*GABAB1R*_ = 0.650; *t*_*GABAB2R*_ = 1.813, *P*_*GABAB2R*_ = 0.144). **I** Quantification of relative levels of proteins as normalized to GAPDH or β-Tubulin protein expression from female cortex at the age of 6 months. *n* = 3 per protein per genotype. One-sample *t-*test (*t*_*D1R*_ = 1.484, *P*_*D1R*_ = 0.211; *t*_*D2R*_ = 6.086, *P*_*D2R*_ = 0.003**; *t*_*D5R*_ = 1.131, *P*_*D5R*_ = 0.321; *t*_*5HT1bR*_ = 1.473, *P*_*5HT1bR*_ = 0.214; *t*_*5HT2aR*_ = 0.231, *P*_*5HT2aR*_ = 0.828; *t*_*5HT2cR*_ = 0.061, *P*_*5HT2cR*_ = 0.954; *t*_*5HT3aR*_ = 0.834, *P*_*5HT3aR*_ = 0.451; *t*_*5HT6R*_ = 0.277, *P*_*5HT6R*_ = 0.794; *t*_*5HT7R*_ = 0.062, *P*_*5HT7R*_ = 0.952; *t*_*GABAB1R*_ = 0.748, *P*_*GABAB1R*_ = 0.495; *t*_*GABAB2R*_ = 1.160, *P*_*GABAB2R*_ = 0.310). **J** None hippocampal CA1 LTD is induced by low-frequency stimulation (LFS) in male control and male *ZFP804A* mutant mice. *n* = 7 cells from 3 mice at the age of 2 months in each group. Student’s *t* test (*t* = 0.3271, *P* = 0.7481). **K** Hippocampal CA1 LTD is induced by LFS in female *ZFP804A* mutant mice, but no in female control mice. *n* = 8–9 cells from 3 mice at the age of 2 months in each group. Student’s *t* test (*t* = 4.245, *P* = 0.0007***). In addition, a significant difference of hippocampal CA1 LTD induced by LFS is also observed between control and *ZFP804A* mutant mice with the genders via two-way ANOVA with Bonferroni correction analysis. Genotyping effect: *F* [1, 30] = 5.081, *P* = 0.0316*; gender factor: *F* [1, 30] = 0.0909, *P* = 0.7651; interaction: *F* [1, 30] = 7.733, *P* = 0.0093**. M male mice, F female mice, Con wild-type controls, Mu *ZFP804A* mutant mice.

It is known that sensorimotor gating, as examined by the PPI test of the acoustic startle response, is impaired in patients with psychiatric disorders including SZ and also in related animal models [46, 47]. We thus examined whether sensorimotor gating was altered in *ZFP804A* mutant mice. No significant differences of PPI were found between control and *ZFP804A* mutant mice in both genders at the age of 2 and 4 months (Supplementary Fig. S4A, and data not shown). However, at 6 months, female *ZFP804A* mutant mice, but not male counterparts, displayed significantly impaired PPI (Supplementary Fig. S4B). Impaired PPI was also observed in 8-month-old female *ZFP804A* mutant mice (Fig. 4A). The results demonstrate that the impairment of sensorimotor gating in *ZFP804A* mutant mice is female-specific and age-dependent.

If this impaired PPI is an SZ-like symptom in the female mutant mice, it could be rescued by atypical antipsychotics, as some of them have been shown to have the association of treatment reponses with *ZNF804A* rs1344706 in SZ patients [48], and are able to rescue the PPI deficiency in rodents [49]. Clozapine, the first developed atypical antipsychotic, binds to multiple receptors, including dopamine, 5-HT, and GABAB receptors to exert its antipsychotic functions [50, 51]. It also appears to have the ability to treat SZ with minimal risk of causing movement disorders, and is indicated for treatment refractory SZ [51]. Thus, we treated 6-month-old female *ZFP804A* mutant mice with clozapine for 2 days and found that the mutant mice indeed showed significant improvement of PPI at 73 and 82 dB to the levels with no significant differences from control mice (Fig. 4B). Next, we examined if there were any differences in the expression of those receptors between control and mutant mice at the age of 6 months when the defects of ZFP804A mutants were apparent in the female mice. Among the receptors examined (D1R, D2R, D5R, 5-HT1bR, 5-HT2aR, 5-HT2cR, 5-HT3aR, 5-HT6R, 5-HT7R, GABAB1R, and GABAB2R), we found that protein levels of 5-HT6R and GABAB1R in the hippocampus were increased, but those of D2R in the cerebral cortex were decreased in the female mutants relative to the age-matched female controls (Fig. 4D, F, G, I). Noteworthy, no obvious changes were detected between male control and male mutant mice (Fig. 4D, E, G, H). These data shed light on the possible mechanism of this sex-specific behavioral phenotype and how clozapine is able to reduce the PPI impairment in *ZFP804A* mutant mice.

Since PPI impairment was observed only in female mice, we suspected that it might be precipitated by estrogen. To address this possibility, 6-month-old female *ZFP804A* mutant mice were ovariectomized, and PPI testing was performed 2 months later. We found that the impaired PPI was absent in ovariectomized mice, with the levels of intensities similar to those of female control mice (Fig. 4C).

The hippocampus is one of the brain regions involved in sensorimotor gating, and hippocampal LTP/LTD is a synaptic model of learning and memory [52, 53]. Therefore, we further examined LTP/LTD induction in hippocampal slices from *ZFP804A* mutant mice and control mice. HFS-induced LTP was comparable between adult *ZFP804A* mutant mice and control mice (Supplementary Fig. S5). It is well known that LFS cannot reliably induce LTD in hippocampal slices from adult wild-type rodents [42, 54, 55], which turned out to be also the case in the present study in control and male *ZFP804A* mutant mice. However, it is notable that LFS induced LTD in hippocampal slices from female *ZFP804A* mutant mice (Fig. 4J, K), indicating that LTD is facilitated in the female mutant mice.

### Reduction of dendritic spine density in cortical and hippocampal neurons of *ZFP804A* mutant mice

*ZNF804A* has been reported to regulate neurite formation and spine density in vitro [25]. Reduction of spine density has been reported in the postmortem brains of SZ patients, as well as in animal models of neuropsychological disorders [41, 56, 57].

**Fig. 5:**
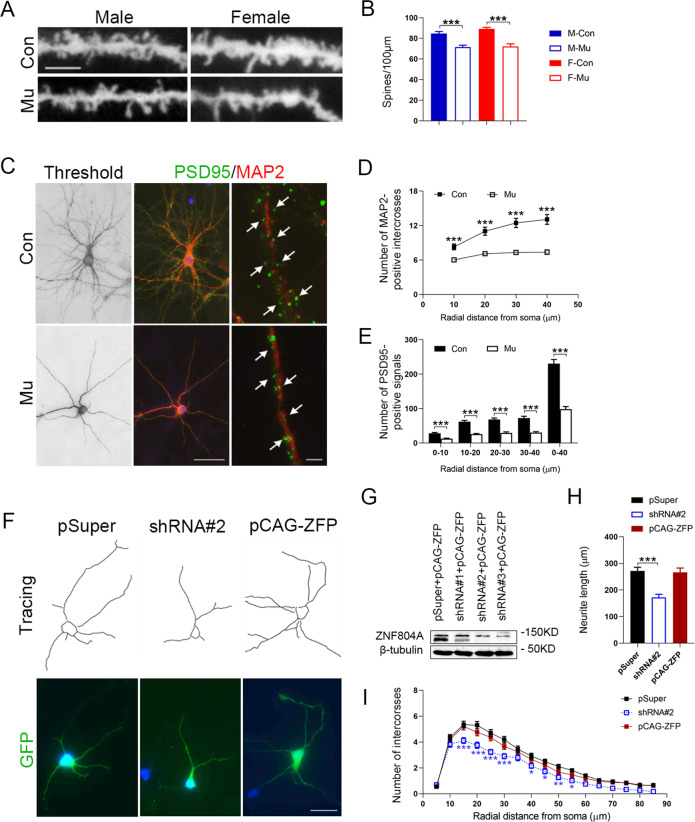
Regulation of dendritic spine density and neurite formation by *ZFP804A* in the hippocampus. **A** Representative images of dendritic spines from hippocampal CA1 areas in mice with indicated genotypes at the age of 8 months. Scale bar = 2.5 μm. **B** Quantification of hippocampal spine density in control and *ZFP804A* mutant mice at this stage. Decreased spine density is seen in *ZFP804A* mutant mice comparing to wild-type mice (*P*_male_ < 0.0001***; *P*_female_ < 0.0001***). *n* = 46–51 in each group. Two-way ANOVA with Bonferroni correction analysis. Genotyping effect: *F* [1, 180] = 59.91, *P* < 0.0001***; sex effect: *F* [1, 180] = 1.750, *P* = 0.1875; interaction: *F* [1, 180] = 1.053, *P* = 0.3063. **C** Representative images of cultured hippocampal neurons immunostained with MAP2 and PSD95 antibodies at DIV 23. Arrowheads indicate PSD95-positive puncta. Scale bars = 50 μm (left and middle four panels), 0.02 μm (right two panels). **D** Significant differences are observed in dendritic branching of cultured hippocampal neurons between wild-type controls and *ZFP804A* mutant mice. *n* = 41 in each group. Student’s *t* test analysis (*t*_10 μm_ = 3.870, *P*_10 μm_ = 0.0002***; *t*_20 μm_ = 5.053, *P*_20 μm_ < 0.0001***; *t*_30 μm_ = 5.940, *P*_30 μm_ < 0.0001***; *t*_40 μm_ = 6.073, *P*_40 μm_ < 0.0001***). **E** Significant differences are observed in PSD95-positive puncta in the dendrites of cultured hippocampal neurons between wild-type controls and *ZFP804A* mutant mice. *n* = 41 in each group. Student’s *t* test analysis (*t*_0–10 μm_ = 5.648, *P*_0–10 μm_ < 0.0001***; *t*_10–20 μm_ = 8.185, *P*_10–20 μm_ < 0.0001***; *t*_20–30 μm_ = 7.244, *P*_20–30 μm_ < 0.0001***; *t*_30–40 μm_ = 6.851, *P*_30–40 μm_ < 0.0001***; *t*_0–40 μm_ = 9.215, *P*_0–40 μm_ < 0.0001***). **F** Representative images of cultured hippocampal neurons transfected with pSUPER, shZFP804A (shRNA#2), or pCAGGS-ZFP804A (pCAG-ZFP) at DIV1 and examined at DIV3. Scale bar = 25 μm. **G** Western blot data show that ShRNA#2 and 3 dramatically reduces the exogenous expression of *ZFP804A* in HEK293T cells. **H** Quantification of GFP-positive neurite length of cultured hippocampal neurons transfected with pSUPER, shRNA#2, or pCAG-ZFP. *n* = 52–70 in each group. One-way ANOVA with Bonferroni correction analysis. Significant differences are found between pSUPER and shRNA#2 groups (*t* = 4.898, *P* < 0.0001***), but not between pSUPER and pCAG-ZFP groups (*t* = 0.2706, *P* > 0.9999). pCAG-ZFP, pCAGGS-ZFP804A. **I** Sholl analysis of neurite branching of cultured hippocampal neurons transfected with pSUPER, shRNA#2, or pCAG-ZFP. *n* = 52–70 in each group. Two-way ANOVA with Bonferroni correction analysis. Transfection effect: *F* [2, 3043] = 63.81, *P* < 0.0001***; radial distance effect: *F* [16, 3043] = 196.2, *P* < 0.0001***; interaction: *F* [32, 3043] = 1.337, *P* = 0.0978. Blue* indicates statistical significance between pSUPER and shRNA#2 groups (*P*_15 μm_ < 0.0001***; *P*_20 μm_ < 0.0001***; *P*_25 μm_ < 0.0001***; *P*_30 μm_ < 0.0001***; *P*_40 μm_ = 0.0187*; *P*_45 μm_ = 0.0181*; *P*_50 μm_ = 0.0081**; *P*_55 μm_ = 0.0129*). F female, M male, Con wild-type controls, Mu *ZFP804A* mutant mice.

Therefore, using Golgi staining, we examined the spine density of pyramidal neurons in the hippocampus and cerebral cortex of *ZFP804A* mutant mice. We counted the number of spines in the hippocampal CA1 region and layers II/III of the frontal cortex because these layers contain more *ZFP804A-*positive neurons (Fig. 1) and the frontal cortex is associated with higher brain functions. In these two regions, the dendritic tree was not noticeably changed (data not shown), but there was a significant reduction of spine density in *ZFP804A* mutant mice comparing to control mice at the age of 8 months (Fig. 5A, B and Supplementary Fig. S6A, B). We observed that the cellular architecture was well maintained in the hippocampus and cerebral cortex of *ZFP804A* mutant mice, as shown by Nissl staining and ISH of layer-specific genes (*Cux2*, *Plxd1*, *Dkk3*) in comparison with wild-type controls (Supplementary Figs. S7 and S8).

The role of *ZFP804A* in regulating spine density was also examined in cultured hippocampal and cortical neurons. Primary cultured neurons were prepared from new-born *ZFP804A* mutant mice, and dendrites were examined at DIV 23. First, Sholl analysis revealed simplification of dendritic arborization: the numbers of intercross along the MAP2-positive dendrites of hippocampal and cortical neurons of *ZFP804A* mutant mice were significantly reduced compared with controls (Fig. 5C, D and Supplementary Fig. S6C, D). PSD95 is localized in postsynaptic sites [58], and we quantified PSD95-positive puncta in the MAP2-positive dendrites, which reveals the presence of spines. The results show that the numbers of PSD95-positive puncta were significantly reduced in cortical and hippocampal neurons of *ZFP804A* mutant mice (Fig. 5E and Supplementary Fig. S6E).

A previous study has demonstrated that knockdown of *ZNF804A* in the human CTX0E16 neural cell line reduces early neurite formation [25]. To see whether this is also the case for mouse neurons, we generated three shRNA constructs all targeting *ZFP804A* and another construct to overexpress *ZFP804A*, which was used to explore the effects of *ZFP804A* overexpression on neurite morphogenesis. The efficacy of *ZFP804A*-shRNA constructs was confirmed by western blot in HEK293 cells (Fig. 5G). One of the *ZFP804A*-shRNA constructs and the overexpression construct were transfected into isolated cortical and hippocampal neurons derived from new-born wild-type mice. The results showed that knockdown of *ZFP804A* caused a significant decrease in neurite branching and length in hippocampal and cortical neurons, whereas overexpression of *ZFP804A* merely induced a dramatic increase of neurite branching and total length in cortical neurons (Fig. 5F, H, I and Supplementary Fig. S6F–H). These data indicate that *ZFP804A* plays a role in regulating neurite morphogenesis in cortical and hippocampal neurons with the difference in the case of overexpression.

## Discussion

GWAS studies showed that SNP variants within *ZNF804A* are associated with mental disorders, especially SZ [7, 9, 12, 13, 59]. Studies of its biological functions in vitro have indicated that *ZNF804A* is involved in cell adhesion, neurite growth, synaptic transmission, and RNA translation, any or all of which may affect brain functions and contribute to the development of psychiatric disorders [5, 6, 24, 25, 27]. We believe that mouse models are needed to further dissect the roles of *ZNF804A* in vivo. By analyzing the *ZFP804A* mutant mice, we found that the mutation does lead to abnormal behaviors including impaired contextual fear memory and spatial memory. Age-dependent sensorimotor gating deficits were also observed, but only in female *ZFP804A* mutants. In addition, abnormal *ZFP804A* expression level induces the decrease of spine density or unusual neurite arborization in the cerebral cortex and hippocampus in vivo and in vitro.

### ZFP804A expression in cortical and hippocampal neurons

Previous studies have reported that *ZFP804A/ZNF804A* is widely expressed in rat and human brain, primarily to neurons [4–6]. Consistent with these reports, our results show that *ZFP804A* mRNA is abundantly distributed in the cerebral cortex and hippocampus of adult mice, particularly in layers II/III and V of the cerebral cortex. By double labeling of *ZFP804A* mRNA and NeuN, we found that nearly all *ZFP804A*-expressing cells were immunostained with NeuN antibody showing that *ZFP804A* is primarily expressed by neurons. In addition, *ZFP804A* is expressed mostly in excitatory neurons, but also in inhibitory neurons. These findings provide the morphological basis for further exploration of *ZFP804A* functions in the brain.

### Sex-independent behavioral deficits of *ZFP804A* mutant mice

Memory impairment is often observed in patients with psychiatric disorders. We found that the acquisition of contextual fear memory was not different between control and *ZFP804A* mutant mice, but that remote contextual fear memory was impaired in *ZFP804A* mutant mice of both genders. During the Morris water maze test training phase, female mutant mice had an increased latency for finding the platform, but male mutant mice showed normal learning ability relative to controls. However, both male and female mutant mice showed obvious deficits in the retrieval of long-term spatial memory. Overall, the data suggest that the mutation of *ZFP804A* affects long-term spatial and contextual fear memories without obvious sex differences.

Previous data from multiple populations have demonstrated that *ZNF804A* is a risk gene for the psychosis [7, 9–13, 59]. Deficits in social interaction may reflect some aspects of SZ’s negative symptoms [60, 61]. However, *ZFP804A* mutant mice display a high preference for animated subject and new stranger mice, just like control mice. Considering the short-time interval between assessments in the three-chamber test, these preferences can be considered as reflection of short-term memory. As such, the social ability and short-term memory appear to be intact but long-term spatial and fear memories are impaired in both sexes of *ZNF804A* mutant mice.

### Female *ZNF804A* mutant mice show age-dependent deficits in sensorimotor gating

The PPI test is widely used to examine sensorimotor gating ability, and PPI deficit correlates to symptoms such as thought disorders and distractibility in SZ patients [62]. We initiated PPI testing in *ZFP804A* mutant mice at the age of 2 and 4 months, and did not detect obvious differences relative to control mice. Deficient PPI was observed at the ages of 6 and 8 months, unexpectedly only in female *ZFP804A* mutant mice. This PPI deficit disappeared in ovariectomized *ZFP804A* mutant mice, which is consistent with estrogen being a major factor affecting sex-specific SZ symptoms [63–67]. Moreover, deficient PPI’s relationship to SZ-like behaviors is strengthened by the fact that clozapine, a well-known atypical anti-SZ agent [50, 51], rescued the phenotype. We also note that the deficient PPI in female *ZFP804A* mutant mice is age-dependent, emerging only after the age of 6 months, in contrast to impairments in spatial and fear memory, which were evident at a younger age. Although we are unaware of any studies reporting age-dependent genetic association of *ZNF804A* variants with SZ, some previous studies have reported that rs7597593 in *ZNF804A* is strongly associated with SZ in women, but not in men [32, 33]. Our findings here further highlight a possible role of *ZNF*804A in sex-dependent SZ phenotypes.

Sex difference also manifests itself in the change of protein levels of two receptors (5-HT6R and GABAB1R) and in LTD facilitation in the hippocampus of the *ZFP804A* mutants. It is generally accepted that therapeutic mechanisms of clozapine in treating SZ are to antagonize the functions of D2R and/or 5-HT2aR [68]. Our data showed that the expression level of 5-HT2aR was not altered in *ZFP804A* mutant mice while that of cortical D2R was decreased. The 5-HT6R is believed to be modulating the functions of multiple neurotransmitter systems in SZ [69], and its expression is indeed increased in the hippocampus of female *ZFP804A* mutant mice. The 5-HT6R is also a target of clozapine, which binds to and antagonizes the activity of the receptor [68] and is used to attenuate PPI deficit in SZ animal models [69, 70]. GABAB1R is the other receptor whose levels were increased in the hippocampus of female mutants. GABAB receptor activity is also reported to be associated with clozapine [50]. GABAB1R is believed to be one of the factors in the regulation of hippocampal LTD, and its abnormality is implicated in the SZ pathophysiology [71, 72]. At present, the answers to whether these female-specific anomalies in the expression of receptors and LTD facilitation are truly interconnected, and how they contribute to the sensorimotor gating deficit are unknown. Further studies are needed to explore any possible underlying mechanisms for the sex differences of *ZFP804A* mutant mice.

### Reduced spine density of hippocampal and cortical neurons in *ZFP804A* mutant mice

Cognitive deficits in many neuropsychiatric disorders are thought to be related to dendritic spines or neurite outgrowth [41, 56, 73, 74]. A recent study has indicated that *ZNF804A* might be involved in neurite formation, maintenance of dendritic spines, and activity-dependent structural plasticity in vitro [25]. Consistent with these findings, our study also revealed a reduction of the density of dendritic spines in cortical and hippocampal neurons of *ZFP804A* mutant mice in vivo and in vitro. This reduction may also be a contributing factor to the impaired spatial and fear memory in both male and female *ZFP804A* mutant mice. However, the dendrite morphology of cortical and hippocampal neurons was not noticeably altered in *ZFP804A* mutant mice in vivo. Therefore the in vitro environment may be affecting the simplified dendrite tree of neurons prepared from *ZFP804A* mutant mice, or some compensation mechanisms may be at work in vivo.

Similar to previous reports [25–27], we found that knockdown of *ZFP804A* impaired the early neurite branching of cortical and hippocampal neurons in vitro. Our data also reveal more roles of *ZNF804A* in regulating neurite morphogenesis: overexpression of *ZNF804A* enhanced neurite branching of cortical neurons in vitro. It is likely that the abnormal expression of *ZFP804A* may be affecting neurite morphogenesis during early developmental stages, which in turn contributes to reduced spine density in adulthood and impaired spatial and fear memory in *ZFP804A* mutant mice. However, it is still unclear what cellular alterations lead to the deficient sensorimotor gating in female *ZFP804A* mutant mice. It is known that LFS cannot induce hippocampal LTD in adult rodents [54, 55], but we found it was able to do so in female *ZFP804A* mutant mice. This result also suggests a possible change of hippocampal synaptic process in female *ZFP804A* mutant mice and some different roles of *ZFP804A* between male and female mice as well.

In brief, we showed that mouse *ZFP804A*, the homolog of human *ZNF804A*, is widely expressed in the cerebral cortex and hippocampus and its transcripts are restricted to neurons. We created *ZFP804A* mutant mice and analyzed their behaviors. Our results indicate that the mutation of *ZFP804A* leads to impaired spatial and fear memory in both genders, and age-dependent deficient sensorimotor gating only in female mice. The sex differences were also observed in the expression level of 5-HT6, GABAB1, and D2 receptors, and hippocampal LTD facilitation. We also found that abnormal *ZFP804A* expression leds to the changes of cortical and hippocampal neurite morphogenesis, including aberrant spine density and neurite arborization. Taken together, these results provide a direct evidence that *ZFP804A/ZNF804A* is associated with deficiencies at behavioral, molecular, and electrophysiological levels like those found in SZ patients. Future detailed studies and analysis on this mouse model may help to gain a better understanding of the role of *ZNF804A* in the adult brain and in nervous system disorders.

## Supplementary information

Supplementary figures
