# Supplementary material for: ZFP804A mutant mice display sex-dependent schizophrenia-like behaviors
Source: Mol Psychiatry. 2020 Dec 10;26(6):2514–32. doi: 10.1038/s41380-020-00972-4 (PMC8440220; doi:10.1038/s41380-020-00972-4)
Supplement: Supplementary file 1 — Supplementary figures [file 41380_2020_972_MOESM1_ESM.pdf]

ZFP804A mutant mice display sex-dependent schizophrenia-like behaviors

Ying Huang, Jing Huang, Qi-Xin Zhou, Chun-Xian Yang, Cui-Ping Yang, Wan-Ying Mei, Lei Zhang, Qiong Zhang, Ling Hu, Yun-Qing Hu, Ning-Ning Song, Sheng-Xi Wu, Lin Xu, Yu-Qiang Ding

Supplementary figures

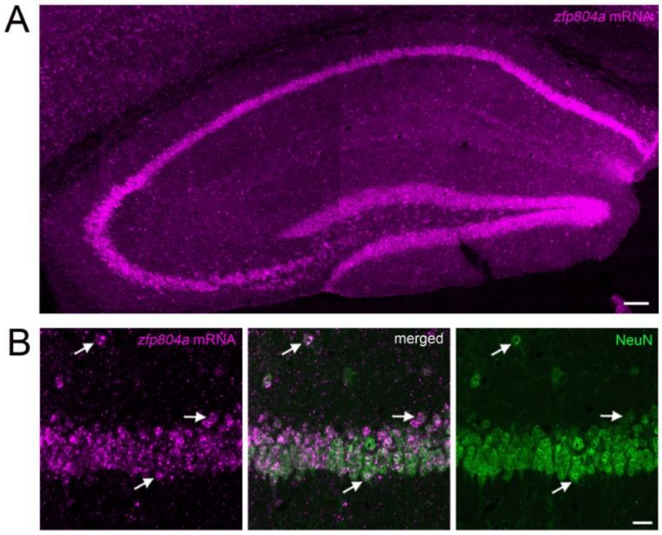

Supplementary Figure S1. Fluorescent ISH shows the distribution of *ZFP804A* mRNA in the hippocampus of adult mouse.

(A) *ZFP804A* mRNA (purple, pseudo-color) is abundantly distributed in hippocampal CA1-3 regions and dentate gyrus.

(B) *Combination* of *in situ* hybridization of *ZFP804A* (purple, pseudo-color) and NeuN immunostaining (green) shows almost all of the *ZFP804A*-expressing cells are immunoreactive for NeuN. Arrows indicate the double-labeled neurons. Scale bars =200 μm (A), and 10 μm (3 magnified panels in B).

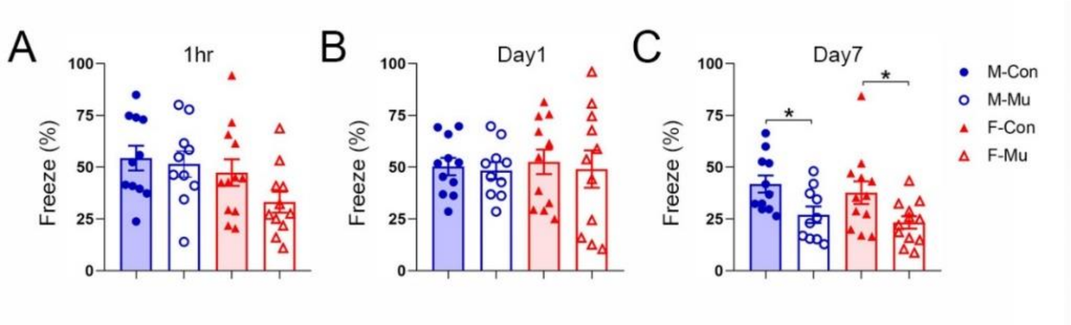

Supplementary Figure S2. The retrieval of contextual fear memory at 1 hour, 1 day and 7 days post fear conditioning in *ZFP804A* mutant mice.

(A) Comparing with wild-type controls, *ZFP804A* mutant mice show similar freezing behaviors 1 hour after fear conditioning (P_{male}=0.9869; P_{female}=0.9869). N=10-12 in each group. Two-way ANOVA with Bonferroni correction analysis. Genotyping effect: F [1, 40]=2.056, P =0.1594; sex effect: F [1, 40]=4.478, P =0.0406* ; interaction: F [1, 40]=0.9027, P =0.3478.

(B) Comparing with wild-type controls mice, *ZFP804A* mutant mice show similar freezing behaviors 1 day after fear conditioning (P_{male}>0.9999; P_{female}>0.9999). N=10-12 in each group. Two-way ANOVA with Bonferroni correction analysis. Genotyping effect: F [1, 40]=3.715x10^{-5}, P =0.9952; sex effect: F [1, 40]=0.1223, P =0.7284; interaction: F [1, 40]=0.0911, P =0.7643.

(C) Comparing with wild-type control mice, reduced freezing behaviors is observed in *ZFP804A* mutant mice 7 days after fear conditioning. N=10-12 in each group. Student's *t*-test analysis (t_{male}=2.558, P_{male}=0.0192*; t_{female}=2.426, P_{female}=0.0239*). Two-way ANOVA with Bonferroni correction analysis. Genotyping effect: F [1, 40]=19.81, P <0.0001***; sex effect: F [1, 40]=0.8612, P =0.359 ; interaction: F [1, 40]=0.0369, P =0.8486. F, female; M, male; C, wild-type controls; Mu, *ZFP804A* mutant mice

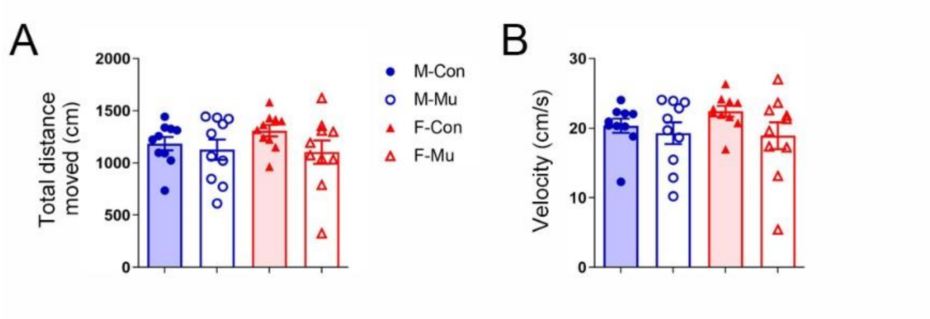

Supplementary Figure S3. Swimming distance and velocity are similar in wild-type control and *ZFP804A* mutant mice.

(A) No significant differences are observed in the total swimming distance between control mice and *ZFP804A* mutant mice (P_{male}>0.9999; P_{female}=0.5886). N=10 in each group. Two-way ANOVA with Bonferroni correction analysis. Genotyping effect: F [1, 36]=2.366, P =0.1327; sex effect: F [1, 36]=0.3477, P =0.5591 ; interaction: F [1, 36]=0.7452, P =0.3937.

(B) No significant differences are observed in the velocity between control mice and *ZFP804A* mutant mice (P_{male}>0.9999; P_{female}=0.5069). N=10 in each group. Two-way ANOVA with Bonferroni correction analysis. Genotyping effect: F [1, 36]=2.675, P =0.1107; sex effect: F [1，36]=0.3666, P =0.5487 ; interaction: F [1, 36]=0.7633, P =0.3881. F, female; M, male; Con, wild-type controls; Mu, *ZFP804A* mutant mice

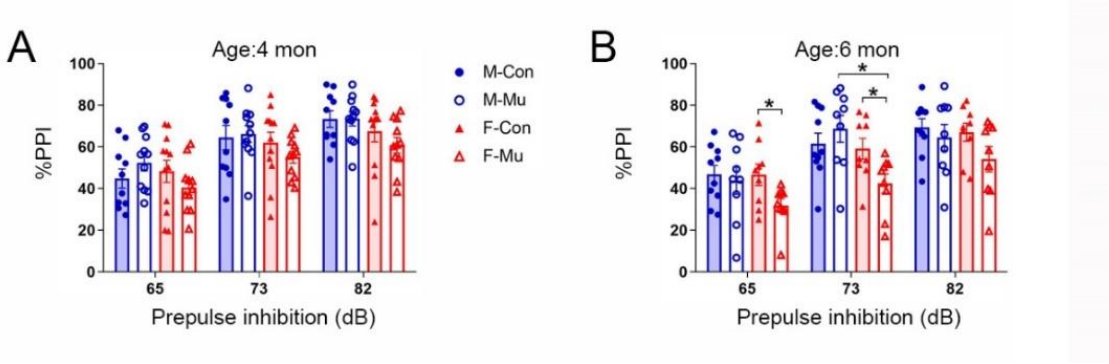

Supplementary Figure S4. PPI responses in *ZFP804A* mutant mice at the age of 4 and 6 months.

(A) Comparing with control mice, *ZFP804A* mutant mice show no differences in PPI test at the age of 4 months. N=10-12 in each group. Two-way ANOVA with Bonferroni correction analysis. At 65 dB, genotyping effect: F [1, 40]=0.0002, P =0.9885; sex effect: F [1, 40]=0.8719, P =0.3560 ; interaction: F [1, 40]=2.915, P =0.0955. At 73 dB, genotyping effect: F [1, 40] =0.3107, P =0.5804; gender factor: F [1, 40] =2.225, P =0.1436; interaction: F [1, 40] =0.8825, P =0.3532. At 82 dB, genotyping effect: F [1, 40] =0.6314, P =0.4315; sex effect: F [1, 40] =4.836, P =0.0337*; interaction: F [1, 40] =0.6744, P =0.4164.

(B) Six-month old female *ZFP804A* mutant mice show lowered PPI value than age-matched female wild-type controls at the prepulse intensity of 65 dB (Student's *t*-test; t_{65dB}=2.442, P_{65dB}=0.0267*) and 73dB (Student's *t*-test; t_{73dB}=2.452, P_{73dB}=0.0261*). N=9-10 in each group. There's also significant difference of PPI value between male and female ZFP804A mutant mice at 73dB (P_{73dB} =0.0101*). Two-way ANOVA with Bonferroni correction analysis. At 73 dB, genotyping effect: F [1, 33] =0.771, P =0.3862; gender factor: F [1, 33] =7.099, P =0.0118*; interaction: F [1, 33] =4.998, P =0.0323*). F, female; M, male; Con, wild-type controls; Mu, *ZFP804A* mutant mice.

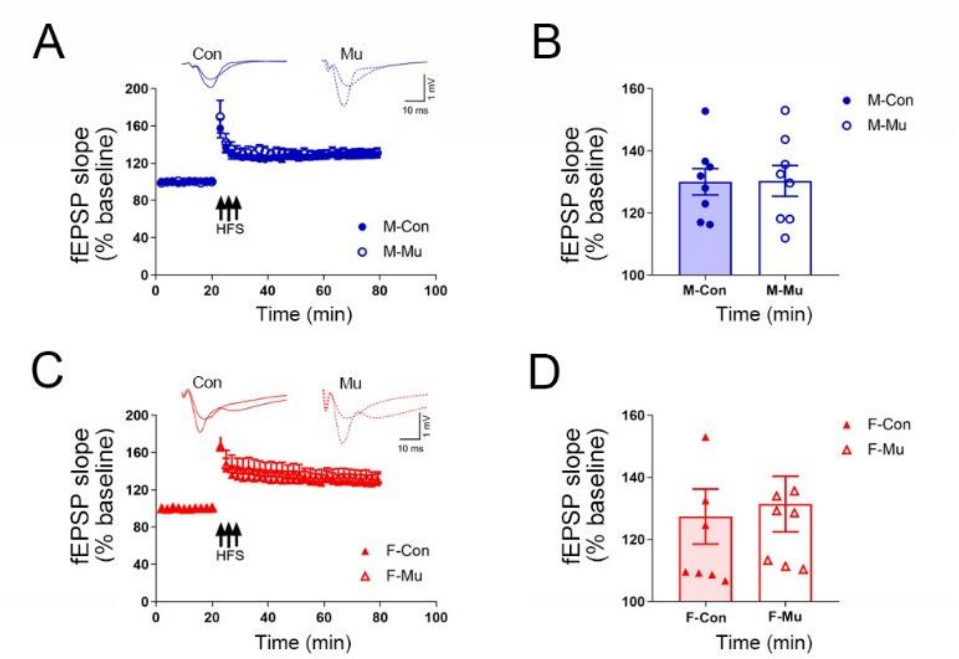

Supplementary Figure S5. Similar hippocampal CA1 LTP measured by fEPSC slope (baseline %) in wild-type controls and *ZFP804A* mutant mice

(A, B) No significant difference of hippocampal CA1 LTP induced by high frequency stimulation (HFS) is observed in male control and *ZFP804A* mutant mice. N=8 cells from 3 mice at the age of 2 months in each group. Student's *t*-test. t=0.0425, P=0.9763.

(C, D) No significant difference of hippocampal CA1 LTP induced by HFS is observed in female controls and *ZFP804A* mutant mice. N=8 cells from 3 mice at the age of 2-mon old in each group. Student's *t*-test. t=0.3179, P=0.7627. F, female; M, male; Con, wild-type controls; Mu, *ZFP804A* mutant mice.

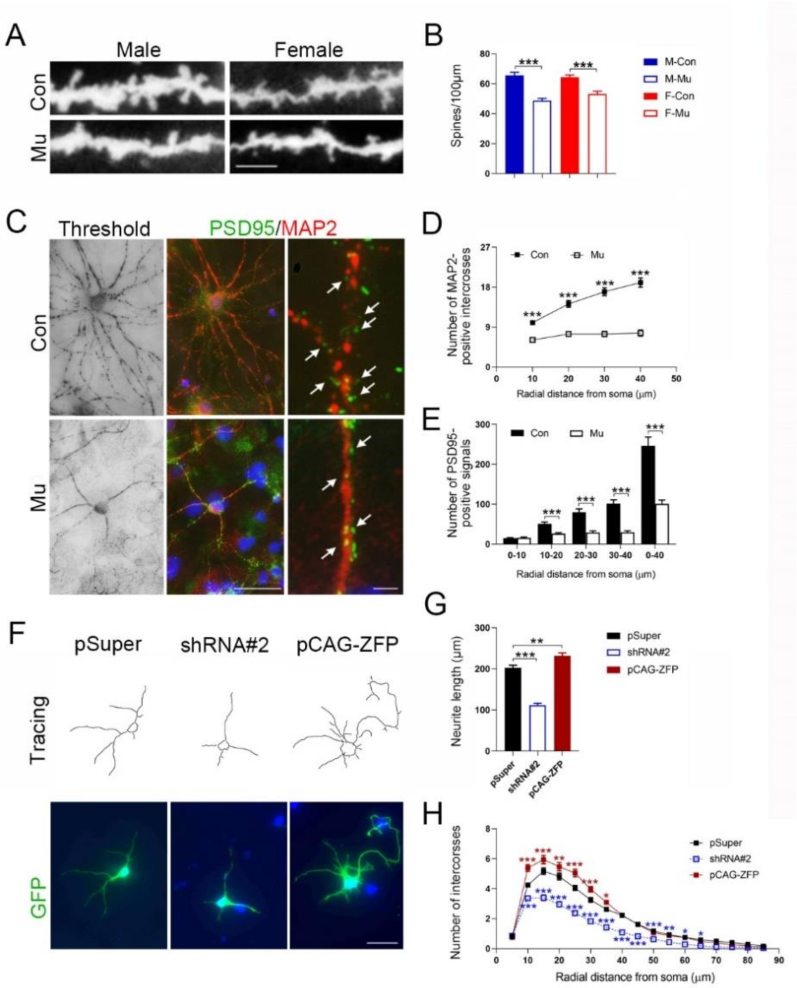

Supplementary Figure S6. Reduced spine density in cortical neurons of *ZFP804A* mutant mice

(A) Representative images of dendritic spines of layer II/III cortical neurons in mice with indicated genotypes at the age of 8 months. Scale bar = 2.5 μm.

(B) Quantification of spine density of the cortical neurons in wild-type controls and *ZFP804A* mutant mice. Decreased spine density was seen in *ZFP804A* mutant mice comparing to WT mice (P_{male} <0.0001***; P_{female} <0.0001***). N=35-36 in each group. Two-way ANOVA with Bonferroni correction analysis. Genotyping effect: F [1, 148]=61.21, P <0.0001***; sex effect: F [1, 148]=0.8387, P =0.3613; interaction: F [1, 148]=2.299, P =0.1316.

(C) Representative images of cultured cortical neurons immunostained with MAP2 and PSD95 antibodies at DIV23. Arrowheads indicate PSD95-positive puncta. Scale bar = 50 μm (left and middle 4 panels), 0.02 μm (right 2 panels).

(D) Significant differences is observed in dendritic branching of the cultured cortical neurons from wild-type controls and *ZFP804A* mutant mice. N=35-36 in each group. Student's *t*-test analysis ($t_{10\mu m}$ =6.176, $P_{10\mu m}$ <0.0001***; $t_{20\mu m}$ =7.180, $P_{20\mu m}$ <0.0001***; $t_{30\mu m}$ =8.614, $P_{30\mu m}$ <0.0001***; $t_{40\mu m}$ =8.799, $P_{40\mu m}$ <0.0001***).

(E) Significant differences are observed in the numbers of PSD95-positive puncta in the dendrites of cultured cortical neurons from the control and *ZFP804A* mutant mice. N=35-36 in each group. Student's *t*-test analysis ($t_{10\text{-}20\mu m}$ =4.420, $P_{10\text{-}20\mu m}$ <0.0001***; $t_{20\text{-}30\mu m}$ =5.425, $P_{20\text{-}30\mu m}$ <0.0001***; $t_{30\text{-}40\mu m}$ =7.127, $P_{30\text{-}40\mu m}$ <0.0001***; $t_{0\text{-}40\mu m}$ =6.137, $P_{0\text{-}40\mu m}$ <0.0001***).

(F) Representative images of cultured cortical neurons transfected with pSUPER, shZFP804A (shRNA#2) or pCAGGS-ZFP804A (pCAG-ZFP) at DIV1 and examined at DIV3. Scale bar = 25 μm.

(G) Quantification of neurite length of cultured cortical neurons transfected with pSUPER, shRNA#2 or pCAG-ZFP. N=89-99 in each group. One-way ANOVA with Bonferroni correction analysis. Significant differences is found between pSUPER and shRNA#2 groups (*t* =10.66, *P* <0.0001***) and between pSUPER and pCAG-ZFP groups (*t* =3.304, *P* =0.0032**).

(H) Sholl analysis of neurite branching of cultured cortical neurons transfected with pSUPER, shRNA#2 or pCAG-ZFP. N=89-99 in each group. Two-way ANOVA with Bonferroni correction analysis. Transfection effect: F [2, 4811]=292.3, P <0.0001***; Radial distance effect: F [16, 4811]=488.3, P <0.0001*** ; interaction: F [32, 4811]=15.01, P <0.0001***. Red* indicates statistical significance between pSUPER and pCAG-ZFP groups. $P_{10\mu m}$ <0.0001***; $P_{15\mu m}$ =0.0001***; $P_{20\mu m}$ =0.0014**; $P_{25\mu m}$ <0.0001***; $P_{30\mu m}$ =0.0004***; $P_{35\mu m}$ =0.0394*. Blue* indicates statistical significance between pSUPER and shRNA#2 groups. $P_{10\mu m}$ <0.0001***; $P_{15\mu m}$ <0.0001***; $P_{20\mu m}$ <0.0001***; $P_{25\mu m}$ <0.0001***; $P_{30\mu m}$ <0.0001***; $P_{35\mu m}$ <0.0001***; $P_{40\mu m}$ <0.0001***; $P_{45\mu m}$ <0.0001***; $P_{50\mu m}$ <0.0001***; $P_{55\mu m}$ =0.0093**; $P_{60\mu m}$ =0.0181*; $P_{65\mu m}$ =0.034*.

F, female; M, male; Con, wild-type controls; Mu, *ZFP804A* mutant mice.

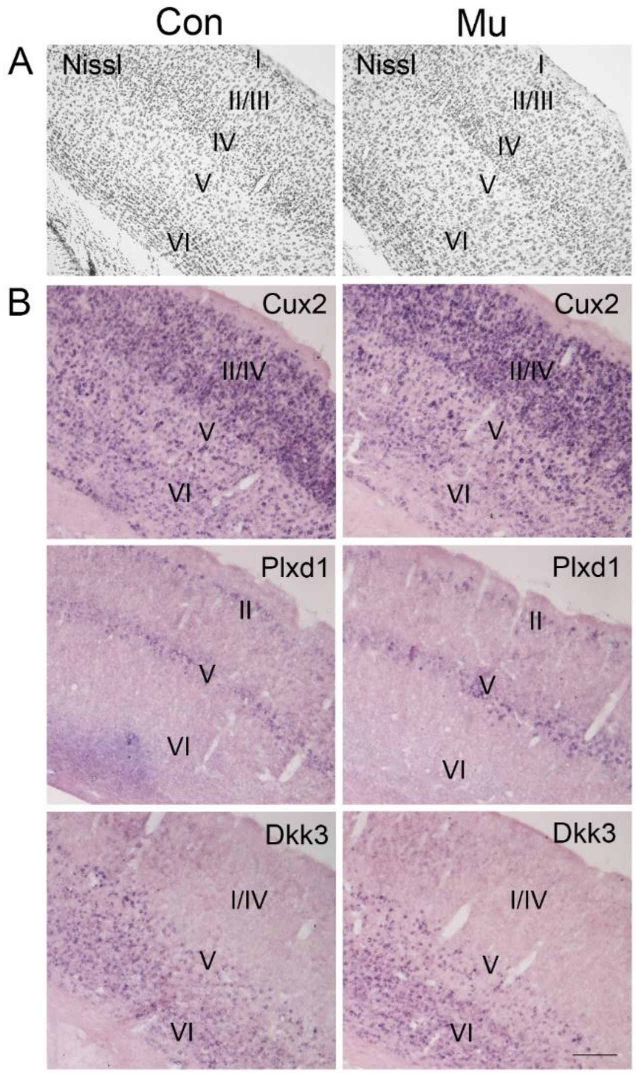

Supplementary Figure S7. Cellular architecture of cortical layers is not obviously altered in *ZFP804A* mutant mice

(A) Representative images of Nissl-stained cerebral cortex at the age of 8 months.

(B) Representative images of ISH of *Cux2*, *Plxd1* and *Dkk3* in the cortex at the age of 8 months. I-VI, cortical layers I-VI; Con, wild-type controls; Mu, *ZFP804A* mutant mice. Scale bar = 150 μm.

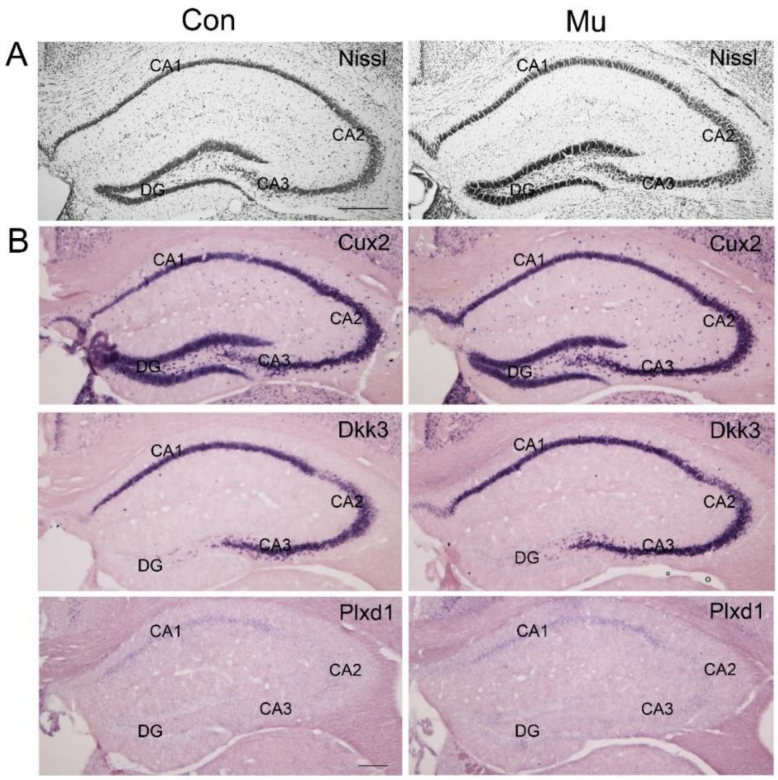

Supplementary Figure S8. Cellular architecture of the hippocampus is not obviously altered in *ZFP804A* mutant mice

(A) Representative images of Nissl-stained hippocampus at the age of 8 months.

(B) Representative images of ISH of *Cux2*, *Dkk3* and *Plxd1* in the hippocampus at the age of 8 months. CA1-3, hippocampal CA1-3 subdivision; DG, dentate gyrus; Con, wild-type controls; Mu, *ZFP804A* mutant mice. Scale bar = 150 μm.
